# Free denoising via overlap measures and *c*-freeness techniques

**DOI:** 10.1007/s00440-026-01507-8

**Published:** 2026-06-13

**Authors:** Maxime Fevrier, Alexandru Nica, Kamil Szpojankowski

**Affiliations:** 1 Laboratoire de mathématiques d’Orsay, Université Paris-Saclay, CNRS, Orsay, 91405 France; 2 Department of Pure Mathematics, University of Waterloo, Waterloo, Ontario Canada; 3 Institute of Mathematics of the Polish Academy of Sciences, ul. Śniadeckich 8, 00–656 Warszawa, Poland; 4 Faculty of Mathematics and Information Science, Warsaw University of Technology, ul. Koszykowa 75, Warszawa, Poland

**Keywords:** 46L54, 60B20, 46L53

## Abstract

We study the problem of *free denoising*. For free selfadjoint random variables *a*, *b*, where we interpret *a* as a signal and *b* as noise, we find $E (a \, | \, a+b)$. To that end, we study a probability measure $\mu ^{( \textrm{ov} )}_{a,a+b}$ on $\mathbb {R} ^2$ which we call the *overlap measure*. We show that $\mu ^{( \textrm{ov} )}_{a,a+b}$ is absolutely continuous with respect to the product measure $\mu _a\times \mu _{a+b}$. The Radon-Nikodym derivative gives direct access to $E (a \, | \, a+b)$. We show that analogous results hold in the case of multiplicative noise when *a*, *b* are positive and the aim is to find $E (a \, | \, a^{1/2}ba^{1/2})$. In a parallel development we show that, for a general selfadjoint expression *P*(*a*, *b*) made with *a* and *b*, finding $E (a \, | \, P(a,b))$ is equivalent to finding the distribution of *P*(*a*, *b*) in a certain two-state probability space $( \mathcal {A} ,\varphi ,\chi )$, where *a*, *b* are c-free with respect to $(\varphi ,\chi )$ in the sense of Bożejko-Leinert-Speicher. We discuss how free denoising (which is set in the framework of an abstract $W^{*}$-probability space) relates to the notion of “matrix denoising” previously discussed in the random matrix literature.

## Introduction

### Problem description

In this paper we consider some instances (primarily the additive instance and the multiplicative instance) of a problem which could go under the name of *free denoising*. The setting for free denoising involves two freely independent selfadjoint random variables *a*, *b* in a tracial $W^{*}$-probability space $\left( \mathcal {A} ,\varphi \right)$, where *a* is viewed as signal (it is “the random variable of interest”), while *b* is viewed as noise. In the additive version of the free denoising problem we assume that the sum a+b is given, and our goal is to find the best approximation of *a* by an element $h(a+b) \in \mathcal {A}$, where *h* is a real-valued Borel function on the spectrum of a+b. The multiplicative version of the problem goes in the same vein, but where *a*, *b* are now assumed to be positive and the task is to approximate *a* by an element $h( \,a^{1/2} b a^{1/2} \, )$. In full generality, the random variable $c \in \mathcal {A}$ which is assumed to be given is some selfadjoint expression c=P(a,b) formed with *a* and *b* (instead of a+b or $a^{1/2} b a^{1/2}$ one could have, for instance, a quadratic expression such as P(a,b)=i(ab-ba) or P(a,b)=ab+ba).

When viewed as an approximation problem with respect to the $|| \cdot ||_2$-norm associated to the trace φ, the above mentioned problem is turned into the one of understanding what is the conditional expectation $E \bigl ( a \mid P(a,b) \bigr )$, where $E \bigl ( \cdot \mid P(a,b) \bigr ): \mathcal {A} \rightarrow \mathcal {B}$ is the unique φ-preserving conditional expectation onto the von Neumann algebra $\mathcal {B} \subseteq \mathcal {A}$ generated by *P*(*a*, *b*). In Sections 6 and 7 of the present paper we provide explicit formulas for such conditional expectations, holding in the two main cases under consideration, $E( a \, | \, a+b)$ and respectively $E \left( a \, | \, a^{1/2} b a^{1/2} \right)$. Then, for a general c=P(a,b), Section 8 of the paper puts into evidence an alternative approach to free denoising which is found by relating it to the notion of “conditionally free independence” of Bożejko-Leinert-Speicher [15]. In the appendix we explain that the methodology described in this paper also works in the case of the product of two freely independent unitaries.

### Motivation: analogies with classical probability

In order to put things into perspective, we note here that free denoising is the free probabilistic counterpart of a well-known formalism of classical probability, concerning conditional expectations (taken in usual “commutative” sense) $\mathbb {E} (X \, | \, Y )$, where (*X*, *Y*) is a random vector in $\mathbb {R} ^2$. Under fairly general conditions (see e.g. the survey paper [17]), the distribution σ of (*X*, *Y*) can be disintegrated with respect to the 1-dimensional distribution ν of *Y*, leading to a family of 1-dimensional distributions $( \mu _t )_{t \in \mathbb {R} }$ such that for a bounded Borel function $f: \mathbb {R} ^2 \rightarrow \mathbb {C}$ we have

1.1 $$\begin{aligned} \int _{ \mathbb {R} ^2} f(s,t) \, d \sigma (s,t) = \int _{ \mathbb {R} } \, \Bigl [ \, \int _{ \mathbb {R} } f(s,t) \, d \mu _t (s) \, \bigr ] \, d \nu (t). \end{aligned}$$

In this setting, one gets that $\mathbb {E} (X \, | \, Y ) = h(Y)$, where we put

1.2 $$\begin{aligned} h(t) = \int _{ \mathbb {R} } s \, d \mu _t (s), \ \ t \in \mathbb {R} . \end{aligned}$$

The preceding formula shows that, in general, the initial data needed in order to find $\mathbb {E} (X \, | \, Y)$ is the full joint distribution σ of *X* and *Y*. But there exist cases when the said conditional expectation can be expressed solely in terms of *Y* and its distribution. To illustrate this, let us focus, in analogy to the previous subsection, on the case where Y=X+Z with *X*, *Z* independent (in the commutative sense) and $Z \sim \mathcal {N}(0,\sigma ^2)$. The distribution of *Y* is then sure to have a density $f_Y$ with respect to Lebesgue measure, and the well-known Tweedie’s formula in statistics (see [26]) says that in this case one has

1.3 $$\begin{aligned} \mathbb {E}(X \, | \, Y) = Y+\sigma ^2 g(Y), \ \text{ where } g(t)=\tfrac{d}{dt} \log (f_Y (t)). \end{aligned}$$

For the free analogue of the above mentioned development, we will use a sibling of the notion of joint distribution which we call “overlap measure” (Section 1.4 below). The methodology developed in connection to this yields, in particular, a free analogue for Tweedie’s formula, which is of the same nature as (1.3). More precisely, for this analogue we pick $a,b \in \mathcal {A}$ to be freely independent and selfadjoint (like at the beginning of Section 1.1 above), and we assume that the distribution of *b* is the Wigner semicircle law with variance $\sigma ^2$. We then find that $E ( a \, | \, a+b)=h(a+b)$ for the function $h: \mathbb {R} \rightarrow \mathbb {R}$ defined by

1.4 $$\begin{aligned} h(t) = t - 2 \pi \, \sigma ^2 \, H_{\mu \boxplus \nu }(t), \end{aligned}$$

where $H_{\mu \boxplus \nu }$ is the Hilbert transform of the measure $\mu \boxplus \nu$. It is remarkable that, analogous to the classical case, the right-hand side of (1.4) depends only on the distribution $\mu \boxplus \nu$ of a+b. The details of the derivation of (1.4) are presented in Example 6.7 below. We mention that this formula had been spotted in the physics literature, via a method called “the replica trick” (see [16]).

A similar phenomenon holds in the multiplicative case, when the noise has free Poisson distribution. We discuss this in Example 1.6 below. As it turns out, this too had been spotted in previous research literature on random matrices, in [21].

### Relation to subordination functions

Let *a*, *b* be freely independent selfadjoint elements in a $W^{*}$-probability space $( \mathcal {A} , \varphi )$. In [13], Biane showed how conditional expectations of the form $E( f(a+b) \, | \, a )$ and $E( g(a^{1/2} b a^{1/2}) \, | \, a )$ can be treated by using the notion of additive and respectively of multiplicative *subordination function* for the Cauchy transforms of the distributions of interest (a precise review of subordination terminology and some statements of results appear in Section 5 below). The development of the present paper makes significant use of subordination functions and of some related Feller-Markov kernels that were identified in [13]; but we are in a certain sense “going in reverse” from what Biane did, since we are looking for $E( f(a) \, | \, a+b )$ rather than $E( f(a+b) \, | \, a )$, and likewise in the multiplicative case. The outcome of the calculations is quite different. For example, as noted by Biane in [13, Remark 3.3], it is always the case that $E( \, (a+b)^n \, | \, a )$ is a polynomial of degree *n* in *a*; in contrast to that, a conditional expectation $E( a^n \, | \, a+b)$ is of the form h(a+b), where *h* (a function on the spectrum of a+b) may not be a polynomial. In fact, Bożejko and Bryc [14] showed that merely assuming $E(a \, | \, a+b)$ and $E(a^2 \, | \, a+b)$ to be linear and respectively quadratic polynomials of a+b forces *a*, *b* to have some special distributions, coming from the free Meixner family.

On the general subject of work which uses subordination and has a topic related to the one of the present paper, we mention here the work [1], where the authors study a problem called “free deconvolution”, which is about retrieving the distribution of *a* when the distribution of a+b and the distribution of the noise *b* are given. Free denoising is different from free deconvolution: it aims to find the element h(a+b) which is at minimal $|| \cdot ||_2$-distance to *a*, and where (as easily seen by computing variance in simple examples with *a*, *b* identically distributed) h(a+b) will generally not have the same distribution as *a*.

### Overlap measure and overlap function

In order to discuss the non-commutative analogue of the disintegration procedure from Equations (1.1), (1.2) of Section 1.2, one replaces the random vector (*X*, *Y*) considered there with a pair of selfadjoint elements *x*, *y* in a $W^{*}$-probability space $( \mathcal {A} , \varphi )$ where the expectation functional φ is assumed to be a faithful trace. The notion of joint distribution of *x* and *y* is usually defined, in this non-commutative setting, as a linear functional $\mu _{x,y}: \mathcal {P} \rightarrow \mathbb {C}$, where $\mathcal {P}$ is the algebra of non-commutative polynomials in two indeterminates and we have $\mu _{x,y}(P)=\varphi (P(x,y))$ for $P \in \mathcal {P}$. For the purpose of finding the conditional expectation $E(x \, | \, y )$, the functional $\mu _{x,y}$ contains too much information; it is convenient to use in its place a bona fide measure $\mu ^{( \textrm{ov} )}_{x,y}$ on the Borel sigma-algebra of $\mathbb {R} ^2$, which we call the *overlap measure* of *x* and *y*, and is defined via the requirement that

$$ \int _{ \mathbb {R} ^2}f(s)g(t) d \mu ^{( \textrm{ov} )}_{x,y} (s,t) = \varphi (f(x)g(y)), \ \ \forall \, f,g \in \textrm{Bor} _b ( \mathbb {R} ), $$

where $\textrm{Bor} _b ( \mathbb {R} )$ is the space complex-valued bounded Borel functions on $\mathbb {R}$.

In the $W^{*}$-framework, the measure $\mu ^{( \textrm{ov} )}_{x,y}$ has been in use for quite some time, and is presented for instance in Section I.1 of [18]. It is worth observing that its construction can be performed more generally, in any ∗-probability space $( \mathcal {A} , \varphi )$ where we have an ability to do Borel functional calculus for selfadjoint elements; we refer to such a structure by calling it a “Borel-ncps”, see Sections 2 and 3 below. Using the Borel-ncps framework is necessary, because it allows one to rigorously consider overlap measures in a ∗-probability space of random N×N matrices (cf. Example 2.9), and to study the weak convergence of such overlap measures in the large *N* limit. Doing so helps explaining why some explicit formulas of free denoisers arising in the $W^{*}$-framework coincide with formulas that had been previously found, via random matrix techniques, in papers about matrix denoising. We comment on this in the final Section 9 of the paper.

The two marginals of $\mu ^{( \textrm{ov} )}_{x,y}$ turn out to be the distributions $\mu _x$ and $\mu _y$ of the two selfadjoint elements *x*, *y* we started with. If $\mu ^{( \textrm{ov} )}_{x,y}$ happens to be absolutely continuous with respect to the direct product $\mu _x \times \mu _y$, then the Radon-Nikodym derivative $d \mu ^{( \textrm{ov} )}_{x,y} / (d \mu _x \times d \mu _y)$ will be referred to as the *overlap function* of *x* and *y*. We note that $\mu ^{( \textrm{ov} )}_{x,y}$ does not always have to be absolutely continuous with respect to $\mu _x \times \mu _y$ (e.g. when y=x, where *x* has absolutely continuous distribution with respect to Lebesgue measure); but this will nevertheless be the case in several relevant examples.

Our motivation for the “overlap” terminology comes from the case when $\mathcal {A} = \mathcal {M} _N ( \mathbb {C} )$, considered with the normalized trace $\varphi = \frac{1}{N} \textrm{Tr}$, when the pair of selfadjoint elements $x,y \in \mathcal {A}$ to be considered is thus a pair of complex Hermitian matrices. In this case, the overlap function ${\textrm{o}}_{x,y}$ is always sure to exist. It is defined on $\textrm{Spec} (x) \times \textrm{Spec} (y)$ where $\textrm{Spec} (x), \textrm{Spec} (y) \subseteq \mathbb {R}$ are the sets of eigenvalues of the matrices *x* and *y*, and its values are computed in terms of quantities $| \langle u,v \rangle |^2$, where $u,v \in \mathbb {C} ^N$ are eigenvectors of *x* and respectively of *y* (cf. Example 3.5 below). For instance: if $\lambda , \rho$ are eigenvalues of multiplicity 1 of *x* and *y*, respectively, and if $u,v \in \mathbb {C} ^N$ are unit vectors such that x(u)=λu and y(v)=ρv, then we get ${\textrm{o}}_{x,y} ( \lambda , \rho ) = N \, | \langle u, v \rangle |^2$ – this is precisely the kind of quantity called an overlap in the physics literature.

The fact that $\mu ^{( \textrm{ov} )}_{x,y}$ is compactly supported on $\textrm{Spec} (x) \times \textrm{Spec} (y)$ holds in general in the $W^{*}$-framework, where $\textrm{Spec} (x), \textrm{Spec} (y) \subseteq \mathbb {R}$ are now the spectra of the selfadjoint elements $x, y \in \mathcal {A}$. As mentioned at the beginning of the subsection, our reason for considering $\mu ^{( \textrm{ov} )}_{x,y}$ in this framework is that it can be used to compute the conditional expectation $E( x \, | \, y )$ via an analogue of the procedure from Equations (1.1), (1.2): disintegrate $\mu ^{( \textrm{ov} )}_{x,y}$ into a family of probability measures $( \mu _t )_{t \in \textrm{Spec} (y)}$ with respect to the distribution of *y* (where every $\mu _t$ is a probability measure on $\textrm{Spec} (x)$), then write $E(x \, | \, y )$ as a function of *y* in the same way as in (1.2), $E( x \, | \, y ) = h(y)$ with $h(t):= \int _{ \textrm{Spec} (x)} s \, d \mu _t (s).$ For the details of how this goes, see Section 4 below, and particularly Proposition 4.3.

### Description of results – additive case

In the case when x=a and y=a+b, with *a*, *b* freely independent selfadjoint random variables, we show that the absolute continuity $\mu ^{( \textrm{ov} )}_{x,y} \ll \mu _x\times \mu _y$ is always sure to hold, and we give an explicit description of the overlap function $d \mu ^{( \textrm{ov} )}_{x,y} / d ( \mu _x\times \mu _y )$. This is obtained by using the relation to subordination functions mentioned above in Section 1.3, and by relying on a known body of results (cf. [4, 5, 7, 11]) about atoms and regularity for a free additive convolution $\mu \boxplus \nu$. The precise description of the result is given as Theorem 1.1 below. The statement of Theorem 1.1.$2^o$ uses some basic structural details of $\mu \boxplus \nu$ – for their review, the reader can have an advanced look at the Section 6 of the paper (and specifically at the “Review 6.2” item over there).

#### Theorem 1.1

Let *a*, *b* be freely independent selfadjoint elements in a $W^{*}$-probability space $( \mathcal {A} , \varphi )$. Let μ and respectively ν be the distributions of *a* and *b* (with respect to φ), and assume that neither of $\mu , \nu$ is a point mass. Note that, due to the free independence of *a* and *b*, the distribution of a+b is equal to $\mu \boxplus \nu$. We let $\omega : \mathbb {C} ^{+} \rightarrow \mathbb {C} ^{+}$ be the subordination function of $\mu \boxplus \nu$ with respect to μ and for every $t \in \mathbb {R}$ we denote by ω(t) (known to exist in $\mathbb {R} \cup \mathbb {C} ^{+} \cup \{ \infty \}$) the limiting value of ω at the point *t*.

$1^o$ Let $\mu ^{( \textrm{ov} )}_{a,a+b}$ be the overlap measure (as discussed in Section 1.4) for the elements *a* and a+b. One has $\mu ^{( \textrm{ov} )}_{a,a+b} \ll \mu \times ( \mu \boxplus \nu )$, and therefore it makes sense to consider the overlap function ${\textrm{o}}_{a,a+b} = d \mu ^{( \textrm{ov} )}_{a,a+b} / d \bigl ( \, \mu \times ( \mu \boxplus \nu ) \, \bigr )$.

$2^o$ Let $\Gamma = \{ \gamma _1, \ldots , \gamma _n \}$ be the (necessarily finite, possibly empty) set of atoms of $\mu \boxplus \nu$, and for every $1 \le i \le n$ consider the decomposition (known to exist and be unique) $\gamma _i = \alpha _i + \beta _i$, with $\alpha _i$ an atom of μ and $\beta _i$ an atom of ν, such that $\mu ( \, \{ \alpha _i \} \, ) + \nu ( \, \{ \beta _i \} \, ) > 1$. On the other hand let $( \mu \boxplus \nu )^{\textrm{ac}}$ denote the absolutely continuous part of $\mu \boxplus \nu$ with respect to Lebesgue measure. It is known that one can write the support of $\mu \boxplus \nu$ (that is, the set $\textrm{Spec} (a+b)$) as a disjoint union $\Gamma \cup U \cup Z$, with $( \mu \boxplus \nu ) (Z) = 0$ and where *U* is a relatively open subset of $\textrm{Spec} (a+b)$, carrying a continuous function $f_{\mu \boxplus \nu }: U \rightarrow (0, \infty )$ which is a version of the Radon-Nikodym derivative $d ( \mu \boxplus \nu )^{\textrm{ac}} (t) / dt$. Moreover, *U* can be picked such that $\omega (t) \in \mathbb {C} ^{+}$, $\forall \, t \in U$.

With the notation introduced above, one can describe the overlap function ${\textrm{o}}_{a,a+b}$ from part $1^o$ by indicating (in a $( \mu \boxplus \nu )$-almost everywhere sense) what are the partial functions ${\textrm{o}}_{a,a+b} ( \cdot , t )$, as follows.

– In the case that $\Gamma \ne \emptyset$, for $t = \gamma _i \in \Gamma$ (with $1 \le i \le n$) one has

1.5 $$\begin{aligned} {\textrm{o}}_{a,a+b} (s, \gamma _i ) = \left\{ \begin{array}{ll} 1/ \mu ( \, \{ \alpha _i \} \, ), &  \text{ if } s = \alpha _i, \\ 0, &  \text{ otherwise } \end{array} \right\} , \ \ s \in \textrm{Spec} (a). \end{aligned}$$

– For t∈U one has

1.6 $$\begin{aligned} {\textrm{o}}_{a,a+b} (s, t) = - \frac{1}{\pi \cdot f_{\mu \boxplus \nu } (t)} \, \textrm{Im} \bigl ( \, \frac{1}{ \omega (t) - s } \, \bigr ), \ \ s \in \textrm{Spec} (a). \end{aligned}$$

Upon combining Theorem 1.1 with a calculation of conditional expectation via disintegration of the overlap measure (as mentioned in Section 1.4), we find:

#### Theorem 1.2

Consider the notation of Theorem 1.1, and let $f: \textrm{Spec} (a) \rightarrow \mathbb {R}$ be a bounded Borel function. One has E[f(a)∣a+b]=h(a+b), where $h: \textrm{Spec} (a+b)\rightarrow \mathbb {R}$ is defined (in $( \mu \boxplus \nu )$-almost everywhere sense) by

1.7 $$\begin{aligned} h(t)={\left\{ \begin{array}{ll} -\frac{1}{\pi }\frac{1}{f_{\mu \boxplus \nu }(t)}\operatorname {Im} \left( \int _{ \mathbb {R} }f(s) \frac{1}{\omega (t)-s} d\mu (s)\right) , &  \text{ if } t\in U, \\ f( \alpha _i ), &  \text{ if } t = \gamma _i, \ 1 \le i \le n. \end{array}\right. } \end{aligned}$$

Moreover, when the function *f* from Theorem 1.2 is f(s)=s, one can further process the formula (1.7), as follows.

#### Proposition 1.3

With the notation of Theorem 1.1, one has $E (a \, | \, a+b)=h(a+b)$ with $h: \textrm{Spec} (a+b)\rightarrow \mathbb {R}$ defined (in $( \mu \boxplus \nu )$-almost everywhere sense) by

1.8 $$\begin{aligned} h(t)={\left\{ \begin{array}{ll} -\frac{\operatorname {Im}\left( \omega (t) \, G_{\mu }( \, \omega (t) \, ) \right) }{\pi \, f_{\mu \boxplus \nu }(t)}, &  \text{ if } t\in U, \\ \alpha _i, &  \text{ if } t = \gamma _i, \ 1 \le i \le n, \end{array}\right. } \end{aligned}$$

where $G_{\mu }: \mathbb {C} ^{+} \rightarrow \mathbb {C} ^{-}$ is the Cauchy transform of μ.

#### Remark 1.4

$1^o$ A relevant fact for our considerations is that, in the setting of Theorem 1.1, the Cauchy transform $G_{\mu \boxplus \nu }: \mathbb {C} ^{+} \rightarrow \mathbb {C} ^{-}$ has continuous extension to $\mathbb {R}$; as a consequence, the subordination relation $G_{\mu \boxplus \nu } (z) = G_{\mu } \bigl ( \, \omega (z) \, \bigr )$, $z \in \mathbb {C} ^{+}$, extends to

1.9 $$\begin{aligned} G_{\mu \boxplus \nu } (t) = G_{\mu } \bigl ( \, \omega (t) \, \bigr ), \ \ t \in U. \end{aligned}$$

Another useful fact concerning the extension of $G_{\mu \boxplus \nu }$ to *U*, which appears in connection to the so-called Stieltjes inversion procedure for $\mu \boxplus \nu$, is that the Radon-Nikodym derivative $f_{\mu \boxplus \nu }$ used in the preceding statements of results can be written as

1.10 $$\begin{aligned} f_{\mu \boxplus \nu } (t) = - \frac{1}{\pi } \, \textrm{Im} \bigl ( \, G_{\mu \boxplus \nu } (t) \, \bigr ), \ \ t \in U. \end{aligned}$$

With these observations, the first branch in Equation (1.8) of the preceding proposition can be put in the form

$$ \frac{\operatorname {Im} \bigl (\omega (t) \, G_{\mu \boxplus \nu }(t) \bigr )}{ \textrm{Im} \bigl ( \, G_{\mu \boxplus \nu }(t) \, \bigr ) }, \ \ t\in U. $$

It is pleasing that, upon doing some parallel considerations on the second branch of (1.8), one can arrive to write an equation of the form

1.11 $$\begin{aligned} h(t) = \lim _{\varepsilon \rightarrow 0^+} \frac{\operatorname {Im} \bigl ( \omega (t + i \varepsilon ) \, G_{\mu \boxplus \nu }(t + i \varepsilon ) \bigr )}{ \textrm{Im} \bigl ( \, G_{\mu \boxplus \nu }(t + i \varepsilon ) \, \bigr ) }, \end{aligned}$$

which covers at the same time the atomic and the absolutely continuous part in the description of *h*(*t*). For the details of how this goes, we refer to Subsection 8.5 below.

$2^o$ In the special case when *b* is a centred semicircular element, Equation (1.8) leads to the free additive analogue of Tweedie’s formula which was announced in Subsection 1.2. The details of how this goes are given in Example 6.7 below.

### Description of results – multiplicative case

Results analogous to those of the preceding subsection hold in connection to the multiplication of two freely independent random variables *a* and *b*, in two cases – when *a*, *b* are assumed to be positive, and respectively when *a*, *b* are assumed to be unitary. We will focus on the positive case, which is presented in this subsection and then detailed in Section 5 below. For the unitary case, a brief exposition of the relevant statements appears in Appendix A at the end of the paper.

Concerning the multiplication of freely independent positive random variables, we prove the following analogue of Theorem 1.1.

#### Theorem 1.5

Let *a*, *b* be freely independent positive random variables in a $W^{*}$-probability space $( \mathcal {A} , \varphi )$. Let μ and respectively ν be the distributions of *a* and *b* with respect to φ, and assume that neither of $\mu , \nu$ is a point mass. Denote by $f_{\mu \boxtimes \nu }$ the density of the absolutely continuous part of $\mu \boxtimes \nu$ and by ω the continuation to the real line of the subordination function of $\mu \boxtimes \nu$ with respect to μ. Then there exists a closed set $0 \in E \subseteq [0, \infty )$, of Lebesgue measure zero, such that

$$\begin{aligned} {\textrm{o}}_{a,a^{1/2}ba^{1/2}} (s,t)= {\left\{ \begin{array}{ll} -\frac{1}{\pi } \frac{1}{tf_{\mu \boxtimes \nu }(t)} \operatorname {Im}\left( \frac{1}{1-\omega (1/t)s}\right) \quad &  \text{ if } s\in \textrm{supp}(\mu )\\ &  \text{ and } t\in \textrm{supp}(\mu \boxtimes \nu )^{ac}\setminus E,\\ \frac{1}{\mu (\{s\})} 1_{\omega (1/t)=1/s}\qquad &  \text{ if } s\in \textrm{supp}(\mu )\\ &  \text{ and } t>0 \text{ is } \text{ an } \text{ atom } \text{ of } \mu \boxtimes \nu ,\\ \frac{1}{\nu (\{0\})}\frac{1}{1-s \psi _\mu ^{-1}(\nu (\{0\})-1)}1_{\nu (\{0\})>\mu (\{0\})} \quad &  \text{ if } s\in \textrm{supp}(\mu )\setminus \{0\}, t=0,\\ \frac{1}{\mu \boxtimes \nu (\{0\})}1_{\mu \boxtimes \nu (\{0\})>0} \qquad &  \text{ if } s=t=0, \end{array}\right. } \end{aligned}$$

defines $\mu \times \mu \boxtimes \nu$-almost everywhere a function ${\textrm{o}}_{a,a^{1/2}ba^{1/2}} : \textrm{Spec} (a)\times \textrm{Spec} (a^{1/2}ba^{1/2})\rightarrow \mathbb {R} _+$. Then $d \mu ^{( \textrm{ov} )}_{a,a^{1/2}ba^{1/2}} (s,t)= {\textrm{o}}_{a,a^{1/2}ba^{1/2}} (s,t) \mu (ds)\,\mu \boxtimes \nu (dt)$. In particular $\mu ^{( \textrm{ov} )}_{a,a^{1/2}ba^{1/2}} \ll \mu \times \mu \boxtimes \nu$ and ${\textrm{o}}_{a,a^{1/2}ba^{1/2}}$ is the overlap function of *a* and $a^{1/2}ba^{1/2}$.

Same as with the description of the overlap function, the general description of conditional expectations $E(f(a)|a^{1/2}ba^{1/2})$ is more involved than in the additive case; we refer to Corollary 7.5 for the precise statement. In the case of f(x)=x we can get a compact form similar to the one in the additive case

$$ h(t)=\lim _{\varepsilon \rightarrow 0^+}\frac{\operatorname {Im}\left( \frac{G_{\mu \boxtimes \nu }(t+i\varepsilon )-\frac{1}{t+i\varepsilon }}{\omega (\frac{1}{t+i\varepsilon })}\right) }{\operatorname {Im}\left( G_{\mu \boxtimes \nu }(t+i\varepsilon )\right) }. $$

We make more comments about this formula in Subsection 8.5.

We next present here the special case when *b* is a free Poisson element.

#### Example 1.6

*(Free Poisson multiplicative noise)* Let *a*, *b* be freely independent positive random variables with respective distributions $\mu , \nu$ in a $W^{*}$-probability space $( \mathcal {A} , \varphi )$. Assume that ν is the free Poisson distribution with parameter λ>0 and, for simplicity, let us also assume that μ has no atom at zero. We then get $E(a \mid a^{1/2}ba^{1/2}) = h(a^{1/2}ba^{1/2})$ with

1.12 $$\begin{aligned} h(t)= {\left\{ \begin{array}{ll} \frac{\lambda t}{|\lambda -1+tG_{\mu \boxtimes \nu }(t)|^2} &  \text{ if } t\in \textrm{supp}(\mu \boxtimes \nu )\setminus \{0\},\\ -\frac{\lambda }{(1-\lambda )G_{(\mu \boxtimes \nu )^{ac}}\left( 0\right) } &  \text{ if } t=0 \text{ when } \lambda <1, \end{array}\right. } \end{aligned}$$

where $G_{\mu \boxtimes \nu }$ and $G_{(\mu \boxtimes \nu )^{ac}}$ are the respective Cauchy transforms of $\mu \boxtimes \nu$ and of its absolutely continuous part. As explained in Section 9 below, this recovers a well-known formula of Ledoit and Péché [21] for the shrinkage estimator of a covariance matrix. Similarly to what was the case for the free additive Tweedie’s formula (1.4), we note here the remarkable fact that, in Equation (1.12), the description of *h* only makes use of the distribution of $a^{1/2}ba^{1/2}$.

### General noise and conditional freeness

In the general case when the noisy element is given by *P*(*a*, *b*) our goal is, once again, to find E(a∣P(a,b)). We show that this problem can be reduced to the problem of finding the distribution of *P*(*a*, *b*) where *a*, *b* are conditionally free (or “c-free”, for short) in the sense of Bożejko-Leinert-Speicher [15].

#### Theorem 1.7

Let *a*, *b* be freely independent selfadjoint non-commutative random variables in a $W^{*}$-probability space $( \mathcal {A} , \varphi )$, where φ is a faithful trace. Let $f:\mathbb {R}\rightarrow \mathbb {R}$ be a bounded non-negative Borel-measurable function such that φ(f(a))=1. Define another state $\chi :\mathcal {A}\rightarrow \mathbb {C}$ by

$$ \chi (y):=\varphi (f(a)y),\qquad \forall y\in \mathcal {A}. $$

Then *a*, *b* are conditionally free with respect to the pair of states $(\varphi ,\chi )$. Moreover, let *P* be a selfadjoint polynomial in two non-commuting variables and denote by $\mu ^{\varphi }_{P(a,b)}$ and $\mu ^{\chi }_{P(a,b)}$ the distributions of *P*(*a*, *b*) with respect to φ and χ, respectively. Then $\mu ^{\chi }_{P(a,b)}\ll \mu ^{\varphi }_{P(a,b)}$ and

$$ E(f(a)\mid P(a,b)) = h \bigl ( \, P(a,b) \, \bigr ) \text{ for } h = \frac{d\mu ^{\chi }_{P(a,b)}}{d\mu ^{\varphi }_{P(a,b)}}. $$

In the setting of Theorem 1.7, the elements *a*, *b* are freely independent with respect to φ, hence $\mu ^{\varphi }_{P(a,b)}$ can be approached by the methods developed in [10] for finding distributions of general polynomials and rational functions in free random variables. Theorem 1.7 brings up the question of understanding, in the *c*-free setting, what is the distribution $\mu ^{\chi }_{P(a,b)}$. We mention that the latter question is currently being investigated in an ongoing work that comes in the continuation of the paper [22]; this is related to the fact that [22] presents a solution to the complementary problem of computing conditional expectations of resolvents $E((z-P(a,b))^{-1}|a)$ (which offers another method for approaching the distribution $\mu ^{\varphi }_{P(a,b)}$).

### Relation to matrix denoising

The problem of free denoising studied in the present paper is a counterpart, set in the framework of a $W^{*}$-probability space, for the statistical problem studied in random matrix theory under the name of *matrix denoising*. In the latter problem, an unknown signal represented by a Hermitian N×N random matrix $A_N$ is corrupted by a noise which is itself a Hermitian N×N random matrix $B_N$, and one would like to “recover the signal” $A_N$ from a noisy observation of the form $P(A_N,B_N)$ – that is, find an estimate of $A_N$ as a function of $P(A_N,B_N)$. Free denoising and matrix denoising are related, due to a well-studied phenomenon of asymptotic free independence for random matrices (see e.g. Chapter 4 of the monograph [23]). This explains, in particular, why the free denoisers from Equations (1.4) and (1.12) are in agreement with formulas that appeared previously in the random matrix literature [16, 21], in papers about matrix denoising. The methods used in these papers are different in scope from those that come up naturally in the $W^{*}$-probability setting (but see, however, the brief discussion about this made in Section 19.4 of the monograph [25]).

Section 9 of the present paper gives a more detailed account of the connection between free denoising and matrix denoising, starting with the general observation that the notion of overlap measure (which also makes sense in a Borel-ncps of N×N random matrices) is well-behaved under the natural notion of convergence in moments for pairs of selfadjoint elements. In a nutshell, the point made in Section 9 is as follows: suppose that a sequence of pairs of Hermitian random matrices $(A_N, B_N)_{N=1}^{\infty }$ converges in moments to a pair (*a*, *b*) of freely independent selfadjoint elements in a $W^{*}$-probability space; then the free denoiser *h* for *a* with respect to *P*(*a*, *b*) provides an asymptotically optimal solution, in $L^2$-sense, to the corresponding matrix denoising problem of $A_N$ with respect to $P(A_N, B_N)$. The term “asymptotically optimal” is considered here with the meaning that no bounded continuous function can asymptotically outperform *h* in the $L^2$-sense; for the precise statements, see Corollary 9.3 in Subsection 9.1, and the specific discussion of additive and multiplicative denoising in Subsections 9.2 and 9.3.

### Organization of the paper

Besides the Introduction, this paper has eight more sections and an Appendix, as follows.

– We introduce and study the overlap measure and overlap function in Section 3, in the setting of a Borel-ncps, after having discussed the Borel-ncps framework in Section 2.

– Starting with Section 4, we focus on the setting of a $W^{*}$-probability space; in particular, Section 4 discusses how, in general, the disintegration of the overlap measure is used to compute conditional expectations $E(x \, | \, y)$ in the $W^{*}$-framework.

– After a review of necessary background in Section 5, we take on additive free denoisers $E( a \, | \, a+b )$ in Section 6, where we detail the results announced in the above Subsection 1.5 of the Introduction. Likewise, in Section 7 we detail the results about multiplicative free denoisers $E( a \, | \, a^{1/2} b a^{1/2} )$ that were announced in Subsection 1.6.

– In Section 8 we consider the case of a general noise (beyond the additive and multiplicative cases), and we explain how free denoising relates to conditional freeness.

– Section 9 is devoted to discussing the relation between free denoising and matrix denoising.

– The paper concludes with an appendix which outlines how the overlap measure approach can be adapted, in the multiplicative case, in connection to the multiplication of free unitaries.

## The framework of a Borel-ncps

### Notation and Remark 2.1

The underlying structure we start from is the one of a ∗-*probability space* $( \mathcal {A} , \varphi )$, with φ a faithful trace. Thus $\mathcal {A}$ is a unital ∗-algebra over $\mathbb {C}$ and $\varphi : \mathcal {A} \rightarrow \mathbb {C}$ is a linear map which is positive and faithful ($\varphi (a^{*}a ) \ge 0$ for all $a \in \mathcal {A}$, with equality if and only if $a = 0_{ \mathcal {A} }$), is tracial ($\varphi (ab) = \varphi (ba)$ for all $a,b \in \mathcal {A}$), and has $\varphi ( 1_{ \mathcal {A} } ) = 1$.

For $( \mathcal {A} , \varphi )$ as above, one considers the set of selfadjoint elements $\mathcal {A} ^{\textrm{sa}}:= \{ x \in \mathcal {A} \mid x = x^{*} \}$, and the set of positive elements

$$ \mathcal {A} ^{+} := \Bigl \{ x \in \mathcal {A} \begin{array}{ll} \vline &  \exists \, k \in \mathbb {N} \text{ and } a_1, \ldots , a_k \in \mathcal {A} \\ \vline &  \text{ such } \text{ that } x = a_1^{*} a_1 + \cdots + a_k^{*} a_k \end{array} \Bigr \}. $$

It is immediate that $\mathcal {A} ^{+}$ is a cone, i.e. it is stable under linear combinations with coefficients in [0,∞), and that $\mathcal {A} ^{\textrm{sa}}$ is the vector space over $\mathbb {R}$ which is generated by $\mathcal {A} ^{+}$ (in the verification of latter fact it is useful to observe that an $x \in \mathcal {A} ^{\textrm{sa}}$ can be written as $x = \frac{1}{4} (x+ 1_{ \mathcal {A} } )^2 - \frac{1}{4} (x- 1_{ \mathcal {A} } )^2$, with $(x \pm 1_{ \mathcal {A} } )^2 \in \mathcal {A} ^{+}$).

The cone $\mathcal {A} ^{+}$ induces a partial order relation on $\mathcal {A} ^{\textrm{sa}}$, where for $x,y \in \mathcal {A} ^{\textrm{sa}}$ one has x≤y if and only if (by definition) $y-x \in \mathcal {A} ^{+}$. We note that the anti-symmetry property of this partial order amounts to $\mathcal {A} ^{+}$ being “pointed” (that is, if *x* and -x are both in $\mathcal {A} ^{+}$, then $x = 0_{ \mathcal {A} }$), where the latter fact is verified by using the faithfulness of φ. We also note that, besides its obvious properties related to addition and scalar multiplication, the partial order on $\mathcal {A} ^{\textrm{sa}}$ has the property that:

2.1 $$\begin{aligned} \text{ If } x \le y \text{ in } \mathcal {A} ^{\textrm{sa}}, \text{ then } \text{ it } \text{ follows } \text{ that } a^{*} x a \le a^{*} y a \text{ in } \mathcal {A} ^{\textrm{sa}} \text{ for } \text{ every } a \in \mathcal {A} . \end{aligned}$$

In order to verify the inequality $a^{*} x a \le a^{*} y a$ claimed in (2.1), one picks $a_1, \ldots , a_k \in \mathcal {A}$ such that $y - x = \sum _{j=1}^k a_j^{*} a_j$ and observes that $a^{*} y a - a^{*} x a = \sum _{j=1}^k (a_j a)^{*} (a_j a) \in \mathcal {A} ^{+}$.

For the purpose of this paper, it is important to be able to invoke a suitable “functional calculus” for an element $x \in \mathcal {A} ^{\textrm{sa}}$. It is evident how that can be done in connection to a polynomial function: if $f: \mathbb {R} \rightarrow \mathbb {C}$ is defined by $f(t) = \alpha _0 + \alpha _1 t + \cdots + \alpha _k t^k$ (with $\alpha _0, \ldots , \alpha _k \in \mathbb {C}$) then for $x \in \mathcal {A} ^{\textrm{sa}}$ we simply put $f(x):= \alpha _0 1_{ \mathcal {A} } + \alpha _1 x + \cdots + \alpha _k x^k \in \mathcal {A}$. But we will need to consider elements $f(x) \in \mathcal {A}$ which are defined for $f: \mathbb {R} \rightarrow \mathbb {C}$ belonging to a larger space of functions, as described in Definition 2.3 below.

### Notation 2.2

We let $\textrm{Bor} ( \mathbb {R} )$ denote the unital ∗-algebra of Borel functions from $\mathbb {R}$ to $\mathbb {C}$. We will work with the unital ∗-subalgebra $\textrm{Bor} _b ( \mathbb {R} ) := \bigl \{ f \in \textrm{Bor} ( \mathbb {R} ) \mid f \text{ is } \text{ bounded } \bigr \},$ and with the slightly larger unital ∗-subalgebra of Borel functions with polynomial growth,

$$ \textrm{Bor}_{\textrm{pol}} ( \mathbb {R} ) = \Bigl \{ f \in \textrm{Bor} ( \mathbb {R} ) \begin{array}{ll} \vline &  \exists \, \alpha , \beta \in [ 0, \infty ) \text{ and } p \in \mathbb {N} \text{ such } \text{ that } \\ \vline &  |f(t)| \le \alpha + \beta \, t^{2p} \text{ for } \text{ all } t \in \mathbb {R} \end{array} \Bigr \} . $$

### Definition 2.3

We will use the name Borel non–commutative probability space abbreviated to *Borel-ncps* for a triple $( \mathcal {A} , \varphi ; \Gamma )$ where: $( \mathcal {A} , \varphi )$ is a ∗-probability space with φ faithful trace, and Γ is a family $\bigl ( \Gamma _x )_{ x \in \mathcal {A} ^{\textrm{sa}} }$ of unital ∗-homomorphisms from $\textrm{Bor}_{\textrm{pol}} ( \mathbb {R} )$ to $\mathcal {A}$, such that the following conditions are fulfillled:

2.2 $$\begin{aligned} \left\{ \begin{array}{l} \text{ Let }\,\text{ id } \in \textrm{Bor}_{\textrm{pol}} ( \mathbb {R} ) \text{ be } \text{ the } \text{ identity } \text{ function, } \textrm{id} (t) = t. \\ \text{ Then } \text{ one } \text{ has: } \Gamma _x ( \textrm{id} ) = x, \ \forall \, x \in \mathcal {A} ^{\textrm{sa}}. \end{array} \right. \end{aligned}$$

2.3 $$\begin{aligned} \left\{ \begin{array}{l} \text{ Let } f,g \text{ and } f_1, f_2, \ldots , f_n, \ldots \text{ in } \textrm{Bor}_{\textrm{pol}} ( \mathbb {R} ) \text{ be } \\ \text{ such } \text{ that } |f_n| \le g \text{ for } \text{ all } n \in \mathbb {N} , \text{ and } \\ \text{ such } \text{ that } \lim _{n \rightarrow \infty } f_n (t) = f(t) \text{ for } \text{ every } t \in \mathbb {R} . \\ \text{ Then } \text{ one } \text{ has: } \lim _{n \rightarrow \infty } \varphi \bigl ( \, x \, \Gamma _y ( f_n ) \, \bigr ) = \varphi \bigl ( \, x \, \Gamma _y (f) \, \bigr ), \ \forall \, x,y \in \mathcal {A} ^{\textrm{sa}}. \end{array} \right. \end{aligned}$$

### Remark 2.4

The way to think about Γ in the preceding definition is that, for $x \in \mathcal {A} ^{\textrm{sa}}$, the unital ∗-homomorphism $\Gamma _x: \textrm{Bor}_{\textrm{pol}} ( \mathbb {R} ) \rightarrow \mathcal {A}$ tells us how to do functional calculus of *x*, with functions from $\textrm{Bor}_{\textrm{pol}} ( \mathbb {R} )$.

We will, in fact, make the following convention of notation:

2.4 $$\begin{aligned} \left\{ \begin{array}{l} \text{ Given } x \in \mathcal {A} ^{\textrm{sa}} \text{ and } f \in \textrm{Bor}_{\textrm{pol}} ( \mathbb {R} ), \\ \text{ we } \text{ use } \text{ the } \text{ notation } f(x) \text{ to } \text{ denote } \text{ the } \text{ image } \Gamma _x (f) \in \mathcal {A} . \end{array} \right. \end{aligned}$$

With this notation, the condition (2.2) takes the form “$\textrm{id} (x) = x$”; together with the homomorphism properties postulated for $\Gamma _x$, this immediately implies that (2.4) extends the formula considered for *f*(*x*) in the last paragraph of Remark 2.1, in the case when *f* is a polynomial function. The ∗-homomorphism properties of $\Gamma _x$ also imply that $f(x) \in \mathcal {A} ^{\textrm{sa}}$ whenever $f \in \textrm{Bor}_{\textrm{pol}} ( \mathbb {R} )$ is real-valued, and that

2.5 $$\begin{aligned} \left( \begin{array}{c} f,g \in \textrm{Bor}_{\textrm{pol}} ( \mathbb {R} ), \text{ real-valued, } \\ \text{ with } f(t) \le g(t) \text{ for } \text{ every } t \in \mathbb {R} \end{array} \right) \ \Rightarrow \ f(x) \le g(x) \end{aligned}$$

(for the latter fact, one writes $g(x) - f(x) = a^{*} a$ with $a = \sqrt{g-f} (x)$).

Let us also record that, in terms of the notation from (2.4), the convergence stated on the last line of (2.3) says that, for functions *f* and $f_n$ as described there, one has

2.6 $$\begin{aligned} \lim _{n \rightarrow \infty } \varphi \bigl ( \, x \, f_n (y) \, \bigr ) = \varphi \bigl ( \, x \, f(y) \, \bigr ), \ \ \forall \, x,y \in \mathcal {A} ^{\textrm{sa}}. \end{aligned}$$

### Remark and Notation 2.5

Let $( \mathcal {A} , \varphi ; \Gamma )$ be a Borel-ncps and let $x \in \mathcal {A} ^{\textrm{sa}}$. The functional calculus for *x* allows us to consider the family of projections $P(t):= \textbf{1}_{]-\infty ,t]}(x) \in \mathcal {A}$, $t \in \mathbb {R}$, and to define a non-decreasing function $F: \mathbb {R} \rightarrow [0,1]$ by $F(t):= \varphi ( \, P(t) \, ), \ t \in \mathbb {R} .$ By using the condition (2.3) from Definition 2.3 one sees that *F* is continuous on the right, with $\lim _{t \rightarrow - \infty } F(t) = 0$ and $\lim _{t \rightarrow \infty } F(t) = 1$. This implies the existence of a Borel probability measure $\mu _x$ on $\mathbb {R}$, uniquely determined, such that *F* is the cumulative distribution function of $\mu _x$. This $\mu _x$ will be referred to as the *distribution of x* with respect to φ. Standard integration arguments show that $\mu _x$ has finite moments of all orders, where for every $n \in \mathbb {N}$ the moment of order *n* of $\mu _x$ is equal to $\varphi (x^n)$. More generally, the functions from $\textrm{Bor}_{\textrm{pol}} ( \mathbb {R} )$ are $\mu _x$-integrable, and one has

2.7 $$\begin{aligned} \int _{ \mathbb {R} } f \, d \mu _x = \varphi \bigl ( \, f(x) \, \bigr ), \ \ \forall \, f \in \textrm{Bor}_{\textrm{pol}} ( \mathbb {R} ). \end{aligned}$$

### Remark and Notation 2.6

Let $( \mathcal {A} , \varphi , \Gamma )$ be a Borel-ncps. We will use the notation “$\textrm{Spec} (a)$” for the spectrum of an $a \in \mathcal {A}$, $\textrm{Spec} (a) := \bigl \{ \lambda \in \mathbb {C} \mid \lambda 1_{ \mathcal {A} } - a \text{ is } \text{ not } \text{ invertible } \bigr \},$ and we note the implication

2.8 $$\begin{aligned} \bigl [ \, x \in \mathcal {A} ^{\textrm{sa}} \, \bigr ] \ \Rightarrow \bigl [ \, \textrm{Spec} (x) \subseteq \mathbb {R} \, \bigr ]. \end{aligned}$$

In other words, (2.8) says that $\lambda 1_{ \mathcal {A} } - x$ is invertible for every $x \in \mathcal {A} ^{\textrm{sa}}$ and $\lambda \in \mathbb {C} \setminus \mathbb {R}$. This holds because we can let $f_{\lambda } \in \textrm{Bor}_{\textrm{pol}} ( \mathbb {R} )$ be defined by $f_{\lambda } (t) = 1/ ( \lambda - t )$, $t \in \mathbb {R}$, and we can use the functional calculus of *x* to get $f_{\lambda } (x) \cdot ( \lambda 1_{ \mathcal {A} } - x )$ $= 1_{ \mathcal {A} } = ( \lambda 1_{ \mathcal {A} } - x ) \cdot f_{\lambda } (x),$ implying the required invertibility of $\lambda 1_{ \mathcal {A} } - x$.

### Example 2.7

Let $( \mathcal {A} , \varphi )$ be a tracial $W^{*}$-probability space, that is, a ∗-probability space where $\mathcal {A}$ is a von Neumann algebra and φ is a normal faithful trace-state. This provides an example of Borel-ncps, where for every $x \in \mathcal {A} ^{\textrm{sa}}$ the ∗-homomorphism $\Gamma _x: \textrm{Bor}_{\textrm{pol}} ( \mathbb {R} ) \rightarrow \mathcal {A}$ is the customary functional calculus for selfadjoint elements (see e.g. [28, Sections I.4 and III.1]). A special feature of this framework is that, for every $x \in \mathcal {A} ^{\textrm{sa}}$, the spectrum $\textrm{Spec} (x)$ is a non-empty compact subset of $\mathbb {R}$, precisely equal to the support of the distribution $\mu _x$ (see e.g. [24, Lecture 3, Proposition 3.15]). For any $f \in \textrm{Bor}_{\textrm{pol}} ( \mathbb {R} )$ and $x \in \mathcal {A} ^{\textrm{sa}}$, the element *f*(*x*) only depends on the restriction of *f* to $\textrm{Spec} (x)$, which is sure to be bounded (which explains why *f*(*x*) comes out as a bounded linear operator, belonging to $\mathcal {A}$).

In preparation of our second main example of Borel-ncps structure, we review the elementary notion of functional calculus for a complex Hermitian matrix.

### Notation and Remark 2.8

Pick $N \in \mathbb {N}$ and let $\mathcal {M} _N^{\textrm{sa}} ( \mathbb {C} ) = \{ X \in \mathcal {M} _N ( \mathbb {C} ) \mid X = X^{*} \}$.

$1^o$ Every function $f: \mathbb {R} \rightarrow \mathbb {C}$ induces a function

2.9 $$\begin{aligned} \Phi _f : \mathcal {M} _N^{\textrm{sa}} ( \mathbb {C} ) \rightarrow \mathcal {M} _N ( \mathbb {C} ), \ \ \Phi _f (X) := f(X), \end{aligned}$$

with “*f*(*X*)” defined in the natural way, $f(X):= \alpha _0 I_N + \alpha _1 X + \cdots + \alpha _k X^k$ where $p(t) = \alpha _0 + \alpha _1 t + \cdots + \alpha _k t^k$ is any polynomial in $\mathbb {C} [t]$ which agrees with *f* on the set of eigenvalues of *X*. Equivalently, one can write $f(X) = U \, f(D) \, U^{*}$, where $X = UDU^{*}$ is any orthogonal diagonalization of *X*, and *f*(*D*) is obtained by applying *f* to the diagonal entries of *D*.

We note the following properties of the function $\Phi _f$ from (2.9):

(i) If $f: \mathbb {R} \rightarrow \mathbb {C}$ is continuous, then $\Phi _f$ is continuous in the norm topology.

(ii) If $f: \mathbb {R} \rightarrow \mathbb {C}$ is a Borel function, then so is $\Phi _f$ (where $\mathcal {M} _N^{\textrm{sa}} ( \mathbb {C} )$ and $\mathcal {M} _N ( \mathbb {C} )$ are

    considered with their natural Borel sigma-algebras).

The verification of (i) is made by uniformly approximating *f* with polynomials on compact intervals of $\mathbb {R}$. Then (ii) follows from (i) and the observation that the algebra of functions $\bigl \{ f \in \textrm{Bor} ( \mathbb {R} ) \mid \Phi _f \text{ is } \text{ Borel } \bigr \}$ is closed under sequential pointwise convergence.

$2^o$ Suppose now that we are also given a probability space $( \Omega , \mathcal {F} , P )$, and let us consider the unital ∗-algebra of matrix-valued random variables

2.10 $$\begin{aligned} L^0 \bigl ( \Omega , \mathcal {M} _N ( \mathbb {C} ) \bigr ) := \Bigl \{ a : \Omega \rightarrow \mathcal {M} _N ( \mathbb {C} ) \begin{array}{ll} \vline &  a \text{ is } \text{ measurable } \text{ between } \mathcal {F} \text{ and } \\ \vline &  \text{ the } \text{ Borel } \text{ sigma-algebra } \text{ of } \mathcal {M} _N ( \mathbb {C} ) \end{array} \Bigr \} \end{aligned}$$

(with random variables *a*, *b* identified when they coincide *P*-almost everywhere). For every $x = x^{*} \in L^0 \bigl ( \Omega , \mathcal {M} _N ( \mathbb {C} ) \bigr )$ and $f \in \textrm{Bor} ( \mathbb {R} )$ we then define $f(x): \Omega \rightarrow \mathcal {M} _N ( \mathbb {C} )$ by

2.11 $$\begin{aligned} \bigl ( \, f(x) \, \bigr ) ( \omega ) := f ( \, x(\omega ) \, ) \in \mathcal {M} _N ( \mathbb {C} ), \ \ \omega \in \Omega . \end{aligned}$$

The function *f*(*x*) defined in (2.11) is measurable, hence is in $L^0 \bigl ( \Omega , \mathcal {M} _N ( \mathbb {C} ) \bigr )$, since $f(x) = \Phi _f \circ x$ with $\Phi _f$ as in (2.9). It is immediate that, for a fixed $x = x^{*} \in L^0 \bigl ( \Omega , \mathcal {M} _N ( \mathbb {C} ) \bigr )$, the functional calculus defined by (2.11) respects the algebraic structure of $\textrm{Bor} ( \mathbb {R} )$, e.g. one has that $(f \, g ) (x) = f(x) \, g(x)$ for any $f,g \in \textrm{Bor} ( \mathbb {R} )$, or that $f(x) \in \bigl [ \, L^0 \bigl ( \Omega , \mathcal {M} _N ( \mathbb {C} ) \bigr ) \, \bigr ]^{+}$ for any real non-negative valued $f \in \textrm{Bor} ( \mathbb {R} )$.

### Example 2.9

We continue to use the framework of the preceding notation, and for every $a \in L^0 \bigl ( \Omega , \mathcal {M} _N ( \mathbb {C} ) \bigr )$ and $1 \le j,k \le N$ we let $\textrm{Entry} _{j,k} (a): \Omega \rightarrow \mathbb {C}$ be the random variable which selects the (*j*, *k*)-entry of *a*. For $a \in L^0 \bigl ( \Omega , \mathcal {M} _N ( \mathbb {C} ) \bigr )$ we will also use the customary notation $\textrm{Tr} (a):= \sum _{j=1}^N \textrm{Entry} _{j,j} (a)$.

The unital ∗-algebra used by this example of Borel-ncps is

2.12 $$\begin{aligned} \mathcal {A} := \bigl \{ a \in L^0 \bigl ( \Omega , \mathcal {M} _N ( \mathbb {C} ) \bigr ) \mid \textrm{Entry} _{j,k} (a) \in L^{\infty -} ( \Omega ) \text{ for } \text{ all } 1 \le j,k \le N \bigr \}, \end{aligned}$$

where $L^{\infty -} ( \Omega )$ is the algebra of complex-valued random variables with finite moments of all orders on $( \Omega , \mathcal {F}, P )$. We note that, thanks to the identity

$$ \textrm{Tr} (a^{*} a ) = \sum _{j,k=1}^N | \textrm{Entry} _{j,k} (a) |^2, \ a \in L^0 \bigl ( \Omega , \mathcal {M} _N ( \mathbb {C} ) \bigr ), $$

one can also describe our algebra $\mathcal {A}$ from (2.12) in the form

2.13 $$\begin{aligned} \mathcal {A} = \bigl \{ a \in L^0 \bigl ( \Omega , \mathcal {M} _N ( \mathbb {C} ) \bigr ) \mid \textrm{Tr} (a^{*} a ) \in L^{\infty -} ( \Omega ) \bigr \}. \end{aligned}$$

The functional $\varphi : \mathcal {A} \rightarrow \mathbb {C}$ that we consider is defined by $\varphi (a) := \frac{1}{N} \, \mathbb {E} ( \, \textrm{Tr} (a) \, ), \ a \in \mathcal {A} .$ It is straightforward to check that in this way we get a ∗-probability space $( \mathcal {A} , \varphi )$ where φ is a faithful trace.

We next observe that the selfadjoint elements of $\mathcal {A}$ have a natural notion of functional calculus, coming from the following fact.

*Fact 1.* Let $x \in \mathcal {A} ^{\textrm{sa}}$ and $f \in \textrm{Bor}_{\textrm{pol}} ( \mathbb {R} )$, and let $f(x) \in L^0 \bigl ( \Omega , \mathcal {M} _N ( \mathbb {C} ) \bigr )$ be defined by (2.11).

      Then $$f(x) \in \mathcal {A} $$.

*Verification of Fact 1.* Since $( \, f(x) \, )^{*} \, f(x) = |f|^2 (x)$, what we must check here (according to (2.13)) is that the non-negative random variable $\textrm{Tr} \bigl ( \, |f|^2 (x) \, \bigr )$ belongs to $L^{\infty -} ( \Omega )$. To that end we pick $\alpha , \beta \in [0, \infty )$ and $p \in \mathbb {N}$ such that $|f|^2 (t) \le \alpha + \beta \, t^{2p}$ for all $t \in \mathbb {R}$, and we use the properties of functional calculus on $L^0 \bigl ( \Omega , \mathcal {M} _N ( \mathbb {C} ) \bigr )$ to find that

2.14 $$\begin{aligned} \textrm{Tr} \bigl ( \, |f|^2 (x) \, \bigr ) \le \textrm{Tr} \bigl ( \, \alpha I_N + \beta x^{2p} \, \bigr ) = N \alpha + \beta \textrm{Tr} ( \, x^{2p} \, ). \end{aligned}$$

The random variable $\textrm{Tr} ( \, x^{2p} \, )$ is in $L^{\infty -} ( \Omega )$, because it can be written as an algebraic expression in the entries $\textrm{Entry} _{j,k} (x) \in L^{\infty -} ( \Omega )$. From the inequality (2.14) it then follows that $\textrm{Tr} \bigl ( \, |f|^2 (x) \, \bigr )$ is in $L^{\infty -} ( \Omega )$ as well. □

It is immediate that, for every $x \in \mathcal {A} ^{\textrm{sa}}$, the assignment f↦f(x) defines a unital ∗-homomorphism $\Gamma _x: \textrm{Bor}_{\textrm{pol}} ( \mathbb {R} ) \rightarrow \mathcal {A}$ such that $\Gamma _x ( \textrm{id} ) = x$. In order to confirm that we are dealing with a Borel-ncps structure, we are thus left to check a dominated convergence condition:

*Fact 2.* The condition (2.3) from Definition 2.3 is satisfied.

*Verification of Fact 2.* Let $( f_n )_{n=1}^{\infty }, f,g \in \textrm{Bor}_{\textrm{pol}} ( \mathbb {R} )$ and $x,y \in \mathcal {A}$ be as in (2.3). We have

2.15 $$\begin{aligned} \varphi \bigl ( \, x \, f_n (y) \, \bigr ) = \frac{1}{N} \int _{\Omega } \textrm{Tr} \bigl ( \, x( \omega ) \, f_n ( y( \omega ) ) \, \bigr ) \, dP( \omega ), \ n \in \mathbb {N} , \end{aligned}$$

and a similar formula holds for $\varphi \bigl ( \, x \, f(y) \, \bigr )$. The pointwise convergence of the $f_n$’s to *f* implies that for every $\omega \in \Omega$ we have $|| \cdot ||-\lim _{n \rightarrow \infty } f_n ( y( \omega ) ) = f( y( \omega ) )$ (norm-convergence in $\mathcal {M} _N ( \mathbb {C} )$), and this immediately entails that $\lim _{n \rightarrow \infty } \textrm{Tr} \bigl ( \, x( \omega ) \, f_n ( y( \omega ) ) \, \bigr ) = \textrm{Tr} \bigl ( \, x( \omega ) \, f( y( \omega ) ) \, \bigr ).$ Thus the integrand on the right-hand side of (2.15) converges pointwise, for $n \rightarrow \infty$, to its counterpart having *f* instead of $f_n$ in its description.

In order to complete the verification of Fact 2, it will thus be sufficient to find a dominating function (independent of *n*) for the integrand on the right-hand side of (2.15) – once this is done, the Lebesgue dominated convergence theorem will give us the desired convergence of $\varphi \bigl ( \, x \, f_n (y)\, \bigr )$ to $\varphi \bigl ( \, x \, f(y) \, \bigr )$.

Towards finding a dominating function, we pick some $\alpha , \beta \in [ 0, \infty )$ and $p \in \mathbb {N}$ such that $g^2 (t) \le \alpha + \beta \, t^{2p}$ for all $t \in \mathbb {R}$; this implies that $|f_n|^2 (t) \le \alpha + \beta \, t^{2p}$ for all $n \in \mathbb {N}$ and $t \in \mathbb {R} ,$ with the further consequence that for every $n \in \mathbb {N}$ and $\omega \in \Omega$ we have

2.16 $$\begin{aligned} \textrm{Tr} \bigl ( \, |f_n|^2 ( y( \omega ) ) \, \bigr ) \le \alpha \, N + \beta \, \textrm{Tr} \bigl ( \, y^{2p} ( \omega ) \, \bigr ) =: v( \omega ). \end{aligned}$$

Returning to the integrand on the right-hand side of (2.15), we then invoke the Cauchy-Schwarz inequality to infer that

2.17 $$\begin{aligned} \vline \ \textrm{Tr} \bigl ( \, x( \omega ) \, f_n ( y( \omega ) ) \, \bigr ) \ \vline \le \sqrt{ u( \omega ) \, v ( \omega ) }, \ \ \forall \, n \in \mathbb {N} \text{ and } \omega \in \Omega , \end{aligned}$$

where v(ω) is defined in (2.16) and we put $u( \omega ):= \textrm{Tr} \bigl ( \, x^2 ( \omega ) \, \bigr )$. We know (from how the algebra $\mathcal {A}$ was defined) that $\textrm{Tr} (x^2), \textrm{Tr} (y^{2p}) \in L^{\infty -} ( \Omega )$, and this immediately implies that $\sqrt{u \, v }$ is in $L^{\infty -} ( \Omega )$ as well. Thus $\sqrt{u \, v }$ is an integrable non-negative random variable which (in view of (2.17)) can be used as our dominating function. □

## Overlap measure and overlap function

In this section we review the construction and some basic properties of a probability measure $\mu ^{( \textrm{ov} )}_{x,y}$ on $\mathbb {R} ^2$, which is associated to a pair of selfadjoint elements *x*, *y* in a Borel-ncps $( \mathcal {A} , \varphi ; \Gamma )$. In the special case when our Borel-ncps is a $W^{*}$-probability space, this construction has been known for a long time, and appears for instance in Section I.1 of [18]. For the reader’s reassurance that the construction extends to the Borel-ncps setting, we provide its details in the proof of Proposition 3.1 below.

The terms “overlap measure” and “overlap function” used in the present paper are inspired from the case when $\mathcal {A} = \mathcal {M} _N ( \mathbb {C} )$ and $\varphi = \frac{1}{N} \textrm{Tr}$. In that case, the description of $\mu ^{( \textrm{ov} )}_{x,y}$ boils down to looking at inner products between the eigenvectors of *x* and those of *y* (cf. Example 3.5 below), and such inner products are known as “overlaps” in the physics and in the random matrix literature.

### Proposition and Definition 3.1

Let $(\mathcal {A},\varphi ; \Gamma )$ be a Borel-ncps, and let $x,y \in \mathcal {A} ^{\textrm{sa}}$. There exists a probability measure μ on the Borel sigma-algebra of $\mathbb {R} ^2$, uniquely determined, such that

3.1 $$\begin{aligned} \int _{ \mathbb {R} ^2}f(s)g(t) d \mu (s,t) = \varphi (f(x)g(y)), \ \ \forall \, f,g \in \textrm{Bor} _b ( \mathbb {R} ), \end{aligned}$$

where the elements $f(x), g(y) \in \mathcal {A}$ that appear in (3.1) are obtained by performing Borel functional calculus on *x* and on *y*, respectively.

We will refer to this probability measure μ as the *overlap measure* of *x* and *y*, and we will denote it as $\mu ^{( \textrm{ov} )}_{x,y}$.

### Proof

*Construction of* μ. We consider the families of projections

$$ P(s) := \textbf{1}_{]-\infty ,s]}(x), \ Q(t) := \textbf{1}_{]-\infty ,t]}(y), \ \ s,t \in \mathbb {R} , $$

and we define $F: \mathbb {R}^2 \rightarrow \mathbb {R}$ by $F(s,t) = \varphi ( \, P(s)Q(t) \, )$, for $s,t \in \mathbb {R}$. We will verify that *F* has the properties required from a cumulative distribution function on $\mathbb {R}^2$.

We start by observing that, due to the trace property of φ, we can write

3.2 $$\begin{aligned} F(s,t) = \varphi \bigl ( \, P(s) \, Q(t) \, P(s) \, \bigr ), \ \ s,t \in \mathbb {R} , \end{aligned}$$

with $P(s) \, Q(t) \, P(s) \in \mathcal {A} ^{+}$. For any fixed $s_o \in \mathbb {R}$, we can invoke (2.5) and then (2.1) in order to find that

$$ 0_{ \mathcal {A} } \le P(s_o) \, Q(t_1) \, P(s_o) \le P(s_o) \, Q(t_2) \, P(s_o) \le P(s_o) \, 1_{ \mathcal {A} } \, P(s_o) = P(s_o), \ \ \forall \, t_1 \le t_2 \in \mathbb {R} ; $$

this, in turn, implies that the function $\mathbb {R} \ni t \mapsto F(s_o,t)$ is non-decreasing, with values in $[0, \varphi ( P(s_o) )].$ A standard calculation based on (2.6) then shows that the latter function is continuous from the right and has $\lim _{t \rightarrow - \infty } F(s_o,t) = 0$, $\lim _{t \rightarrow \infty } F(s_o,t) = \varphi ( P(s_o) ).$

Likewise, one sees that for fixed $t_o \in \mathbb {R}$ the function $\mathbb {R} \ni s \mapsto F(s,t_o)$ is non-decreasing and continuous from the right, with $\lim _{s \rightarrow - \infty } F(s,t_o) = 0$, $\lim _{s \rightarrow \infty } F(s,t_o) = \varphi ( Q(t_o) ).$ Arguments similar to the above show, moreover, that *F* has correct limits (of 0 or 1) when both $s, t \rightarrow \pm \infty$.

In order to argue that *F* is a 2-dimensional cumulative function, we are left to verify that for every $s_1 < s_2$ and $t_1 < t_2$ in $\mathbb {R}$ we have

3.3 $$\begin{aligned} F(s_2,t_2) - F(s_2,t_1) - F(s_1,t_2) + F(s_1,t_1) \ge 0. \end{aligned}$$

The reader should have no difficulty to perform the bit of linear algebra which rewrites the quantity from (3.3) in the form $\varphi \bigl ( \, (P(s_2) - P(s_1)) \, (Q(t_2) - Q(t_1)) \, (P(s_2) - P(s_1) \, \bigr )$; the latter quantity is indeed sure to be ≥0, since φ is applied there to an element from $\mathcal {A} ^{+}$.

As a consequence of the above, we infer the existence of a probability measure μ on the Borel sigma-algebra of $\mathbb {R} ^2$, such that $\mu \bigl ( \, ] - \infty , s] \, \times \, ] - \infty , t] \, \bigr ) = F(s,t), \ \ \forall \, s,t \in \mathbb {R} .$

*Verification that* (3.1) *holds.* The measure μ constructed above has the property that

3.4 $$\begin{aligned} \mu \bigl ( \, ] s_1, s_2] \, \times \, ] t_1, t_2] \, \bigr ) = \varphi \bigl ( \, \textbf{1}_{ ] s_1, s_2] } (x) \, \textbf{1}_{ ] t_1, t_2] } (y) \, \bigr ), \ \ \forall \, s_1< s_2 \text{ and } t_1 < t_2 \text{ in } \mathbb {R} ; \end{aligned}$$

this holds because, as immediately verified, both sides of (3.4) are equal to the algebraic expression considered in (3.3).

In order to upgrade (3.4) to the formula indicated in (3.1), we use an intermediate step, as follows: for every $s_1 < s_2$ in $\mathbb {R}$ we put

$$ \mathcal {V} _{s_1,s_2} := \{ g \in \textrm{Bor}_b ( \mathbb {R} ) \mid \text{(3.1) } \text{ holds } \text{ for } \textbf{1}_{ ]s_1, s_2] } \text{ and } g \}, $$

and (by invoking appropriate features of the Borel functional calculus for the element *y*) we observe that $\mathcal {V} _{s_1, s_2}$ is a linear subspace of $\textrm{Bor}_b ( \mathbb {R} )$ which is closed under the pointwise convergence of uniformly bounded sequences of functions. But let us also observe that, as a consequence of (3.4), the space $\mathcal {V} _{s_1, s_2}$ contains the indicator functions of all the half-open intervals $] t_1, t_2]$ with $t_1 < t_2$ in $\mathbb {R}$. Together with stability under linear combinations and under uniformly bounded pointwise convergence, this implies that $\mathcal {V} _{s_1, s_2} = \textrm{Bor}_b ( \mathbb {R} )$.

Now, for every $g \in \textrm{Bor}_b ( \mathbb {R} )$ let us put $\mathcal {W} _g := \{ f \in \textrm{Bor}_b ( \mathbb {R} ) \mid \text{(3.1) } \text{ holds } \text{ for } f \text{ and } g \}.$ The intermediate step taken in the preceding paragraph assures us that $\mathcal {W} _g$ contains the indicator functions of all the half-open intervals $] s_1, s_2]$ with $s_1 < s_2$ in $\mathbb {R}$. On the other hand, $\mathcal {W} _g$ is found (by invoking appropriate features of the Borel functional calculus for the element *x*) to be a linear subspace of $\textrm{Bor}_b ( \mathbb {R} )$ which is closed under the pointwise convergence of uniformly bounded sequences of functions. Putting together all these properties forces the conclusion that $\mathcal {W} _g = \textrm{Bor}_b ( \mathbb {R} )$.

Finally: the equality $\mathcal {W} _g = \textrm{Bor} _b ( \mathbb {R} )$, holding for all $g \in \textrm{Bor} _b ( \mathbb {R} )$, amounts precisely to the fact that the required Equation (3.1) holds for all $f,g \in \textrm{Bor}_b ( \mathbb {R} )$.

*Uniqueness of* μ. Let ν be a probability measure on $\mathbb {R} ^2$ which has the same property as described for μ in (3.1). For any two Borel sets $A,B \subseteq \mathbb {R}$ we then find that

$$ \nu (A \times B) = \varphi \bigl ( \, \textbf{1}_A (x) \, \textbf{1}_B (y) \, \bigr ) = \mu (A \times B). $$

The measures μ and ν thus agree on the collection of sets $\{ A \times B \mid A,B \text{ Borel } \text{ subsets } \text{ of } \mathbb {R} \}$. Since the latter collection is a π-system which generates the Borel sigma-algebra of $\mathbb {R} ^2$, we conclude that $\nu = \mu$. □

### Remark 3.2

$1^o$ In the preceding proposition it was convenient to only state Equation (3.1) for $f,g \in \textrm{Bor} _b ( \mathbb {R} )$, as that does not require any discussion concerning the integrability of the function (s,t)↦f(s)g(t) on the left-hand side. We leave it as an exercise to the reader to check that, due to how the framework of a Borel-ncps was set in Section 2, the equality stated in (3.1) continues to make sense and to hold true for functions $f,g \in \textrm{Bor}_{\textrm{pol}} ( \mathbb {R} )$ – that is, the function (s,t)↦f(s)g(t) is $\mu ^{( \textrm{ov} )}_{x,y}$-integrable, and its integral against $\mu ^{( \textrm{ov} )}_{x,y}$ is equal to $\varphi \bigl ( \, f(x) \, g(y) \, \bigr )$.

$2^o$ When in Equation (3.1) we set the function *g* to be identically equal to 1, we find that the first one-dimensional marginal of $\mu ^{( \textrm{ov} )}_{x,y}$ (that is, the Borel probability measure on $\mathbb {R}$ defined by $A \mapsto \mu ^{( \textrm{ov} )}_{x,y} ( A \times \mathbb {R} )$) coincides with the distribution $\mu _x$ of the element *x* with respect to φ, as defined in Section 2 (cf. Remark and Notation 2.5). A similar argument shows that the second marginal of $\mu ^{( \textrm{ov} )}_{x,y}$ is equal to $\mu _y$, the distribution of *y* with respect to φ.

### Definition 3.3

Consider the same framework and notation as above and suppose that the overlap measure $\mu ^{( \textrm{ov} )}_{x,y}$ is absolutely continuous with respect to the direct product of its marginals $\mu _x$ and $\mu _y$. The Radon-Nikodym derivative

3.5 $$\begin{aligned} {\textrm{o}}_{x,y} := \frac{d \mu ^{( \textrm{ov} )}_{x,y} }{d ( \mu _x \times \mu _y )} \end{aligned}$$

will be referred to as the *overlap function* of *x* and *y*.

### Example 3.4

In the setting of Proposition and Definition 3.1, consider the situation where *x* and *y* are independent, either in the classical (commutative) sense, or in the sense of free probability. This assumption has the consequence that one can factor

$$ \varphi \bigl ( \, f(x) \, g(y) \, \bigr ) = \varphi \bigl ( \, f(x) \, \bigr ) \cdot \varphi \bigl ( \, g(y) \, \bigr ), \ \ \forall \, f,g \in \textrm{Bor} _b ( \mathbb {R} ). $$

The latter equality can be read as saying that

$$ \int _{ \mathbb {R} ^2} f(s) \, g(t) \, d \mu ^{( \textrm{ov} )}_{x,y} (s,t) = \int _{ \mathbb {R} } f(s) \, d \mu _x (s) \cdot \int _{ \mathbb {R} } g(t) \, d \mu _y (t), $$

which entails that the overlap measure $\mu ^{( \textrm{ov} )}_{x,y}$ is equal to the direct product $\mu _x \times \mu _y$. In this case, the overlap function ${\textrm{o}}_{x,y}$ can be taken as the constant function one.

### Example 3.5

Consider the situation where our Borel-ncps $( \mathcal {A} , \varphi )$ has $\mathcal {A} = \mathcal {M} _N ( \mathbb {C} )$, with $\varphi = \textrm{tr}_N$ (normalized trace), and where the elements of $\mathcal {A} ^{\textrm{sa}}$ are thus N×N Hermitian matrices. Let us pick some $x,y \in \mathcal {A} ^{\textrm{sa}}$, and let $\lambda _1, \ldots , \lambda _N$ and $\rho _1, \ldots , \rho _N$ be the eigenvalues of *x* and respectively of *y*, counted with multiplicities. Moreover, let us consider two orthonormal bases $u_1, \ldots , u_N$ and $v_1, \ldots , v_N$ for $\mathbb {C} ^N$, such that $x (u_k) = \lambda _k u_k$ and $y (v_k) = \rho _k v_k$ for $1 \le k \le N$. Some elementary linear algebra calculations show that, in this situation, the overlap measure of *x* and *y* is

$$ \mu ^{( \textrm{ov} )}_{x,y} = \sum _{k, \ell = 1}^N \frac{ \mid \langle u_k \, , \, v_{\ell } \rangle \mid ^2 }{N} \, \delta _{( \lambda _k, \rho _{\ell } )} $$

(where $\delta _{( \lambda , \rho )}$ stands for the Dirac mass concentrated at the point $(\lambda , \rho ) \in \mathbb {R} ^2$).

We note that in this example we have

$$ \mu _x \times \mu _y = \Bigl ( \, \frac{1}{N} \sum _{k=1}^N \delta _{\lambda _k} \, \Bigr ) \times \Bigl ( \, \frac{1}{N} \sum _{k=1}^N \delta _{\rho _k} \, \Bigr ) = \frac{1}{N^2} \sum _{k, \ell =1}^N \delta _{ ( \lambda _k, \rho _{\ell } ) }, $$

which makes it clear that $\mu ^{( \textrm{ov} )}_{x,y} \ll \mu _x \times \mu _y$ (indeed, absolute continuity simply amounts here to the fact that the set of atoms of $\mu ^{( \textrm{ov} )}_{x,y}$ is contained in the set of atoms of $\mu _x \times \mu _y$). Given eigenvalues λ of *x* and ρ of *y*, the value at $( \lambda , \rho )$ of the overlap function ${\textrm{o}}_{x,y}$ is the ratio $\mu ^{( \textrm{ov} )}_{x,y} \bigl ( \, \{ \, ( \lambda , \rho ) \, \} \, \bigr )/\Bigl ( \mu _x ( \, \{\lambda \} \, ) \cdot \mu _y ( \, \{ \rho \} \, ) \Bigr ).$ An easy application of Parseval’s formula shows that the latter quantity can be also written as

$$ {\textrm{o}}_{x,y} ( \lambda , \rho ) = N \, \frac{\textrm{Tr} (p \, q)}{\textrm{Tr}(p) \cdot \textrm{Tr} (q) }, $$

where *p* is the projection onto the eigenspace of *x* corresponding to λ, and *q* is the projection onto the eigenspace of *y* corresponding to ρ.

## Conditional expectations, via disintegration of the overlap measure

In this section we fix a $W^{*}$-probability space $( \mathcal {A} , \varphi )$, as considered in Example 2.7, and we discuss some additional structure that appears in this setting.

### Remark and Notation 4.1

As mentioned in Example 2.7, the spectrum $\textrm{Spec} (x)$ of an $x \in \mathcal {A} ^{\textrm{sa}}$ is a non-empty compact subset of $\mathbb {R}$, and the support of the distribution $\mu _x$ is precisely equal to $\textrm{Spec} (x)$.

Let us now simultaneously consider two elements $x,y \in \mathcal {A} ^{\textrm{sa}}$, and their overlap measure $\mu ^{( \textrm{ov} )}_{x,y}$. The observation recorded in the preceding paragraph entails that $\mu ^{( \textrm{ov} )}_{x,y}$ has compact support, contained in $\textrm{Spec} (x) \times \textrm{Spec} (y)$ (the Cartesian product of the supports of the two marginals $\mu _x$ and $\mu _y$ of $\mu ^{( \textrm{ov} )}_{x,y}$). In connection to that, an important piece of structure which will be consistently used in what follows is that $\mu ^{( \textrm{ov} )}_{x,y}$ can be *disintegrated* with respect to either of its marginals $\mu _x$ or $\mu _y$ (see e.g. [19, Chapter III, pages 78-79]). For instance, the disintegration with respect to $\mu _y$ comes in the guise of a family $\bigl \{ k_t^{(2)} \mid t \in \textrm{Spec} (y) \bigr \}$, where every $k_t^{(2)}$ is a probability measure on the Borel sigma-algebra of $\textrm{Spec} (x)$, such that the following things happen. First, for every bounded Borel measurable function $f: \textrm{Spec} (x) \rightarrow \mathbb {C}$, the “fiber-wise integration of *f*”,

4.1 $$\begin{aligned} t \mapsto \int _{ \textrm{Spec} (x)} f(s) \, d k_t^{(2)} (s) \text{ for } t \in \textrm{Spec} (y), \end{aligned}$$

is a bounded Borel measurable function on $\textrm{Spec} (y)$. Then, for every pair of bounded Borel measurable functions $f: \textrm{Spec} (x) \rightarrow \mathbb {C}$ and $g: \textrm{Spec} (y) \rightarrow \mathbb {C}$, one has that

4.2 $$\begin{aligned} \int _{ \textrm{Spec} (y)} \Bigl [ \, \int _{ \textrm{Spec} (x)} f(s) \, d k_t^{(2)} (s) \, \Bigr ] \, g(t) \, d \mu _y (t) = \int _{ \textrm{Spec} (x) \times \textrm{Spec} (y) } f(s)g(t) \, d \mu ^{( \textrm{ov} )}_{x,y} (s,t). \end{aligned}$$

Likewise, disintegration of $\mu ^{( \textrm{ov} )}_{x,y}$ with respect to $\mu _x$ creates a family $\bigl \{ k_s^{(1)} \mid s \in \textrm{Spec} (x) \bigr \}$, where every $k_s^{(1)}$ is a probability measure on the Borel sigma-algebra of $\textrm{Spec} (y)$, and where formulas symmetric to (4.1) and (4.2) are holding.

Another relevant piece of structure that we are sure to have for the $( \mathcal {A} , \varphi )$ of this section concerns conditional expectations.

### Remark and Notation 4.2

For every von Neumann subalgebra $\mathcal {B} \subseteq \mathcal {A}$ one has a φ-preserving *conditional expectation* onto $\mathcal {B}$, which will be denoted as $E( \cdot | \mathcal {B} )$; this is the linear map from $\mathcal {A}$ to $\mathcal {B}$ uniquely determined by the requirement that

4.3 $$\begin{aligned} \varphi \bigl ( \, E(a |\mathcal {B}) \, b \, \bigr ) = \varphi (ab), \ \ \forall \, a\in \mathcal {A} \text{ and } b\in \mathcal {B} . \end{aligned}$$

We note that $E( a | \mathcal {B} )$ is a non-commutative analogue for the classical conditional expectation $E(X|\mathcal {G})$ where *X* is a bounded complex random variable on a probability space $( \Omega , \mathcal {F} , \mathbb {P} )$ and $\mathcal {G}$ is a sub-σ-algebra of $\mathcal {F}$. The commutative case is retrieved as the special case of (4.3) where $\mathcal {A} = L^{\infty } ( \Omega , \mathcal {F} , \mathbb {P} )$, with $\varphi : \mathcal {A} \rightarrow \mathbb {C}$ being the standard expectation functional, and where one lets $\mathcal {B}:=L^{\infty }( \Omega , \mathcal {G},\mathbb {P}) \subseteq \mathcal {A}$.

From (4.3) it is immediate that the map $E( \cdot | \mathcal {B} )$ is a projection (that is, $E(b | \mathcal {B} ) = b$ for $b \in \mathcal {B}$), and indeed has the “conditional expectation property” (a.k.a. $\mathcal {B}$-bimodule property) that $E(b_1 a b_2 | \mathcal {B} ) = b_1 \, E(a | \mathcal {B} ) \, b_2$ for all $a \in \mathcal {A}$ and $b_1, b_2 \in \mathcal {B}$. $E( \cdot | \mathcal {B} )$ is, moreover, a normal, positive and faithful map (see e.g. [28, Section V.2]); its positivity also implies, in particular, that $E(a| \mathcal {B} )$ is selfadjoint in $\mathcal {B}$ whenever *a* is selfadjoint in $\mathcal {A}$. For the present paper, a relevant property of $E( \cdot | \mathcal {B} )$ (easily derived from (4.3)) is that it serves as orthogonal projection in the $L^2$-space associated to the trace φ; thus, for every $a \in \mathcal {A}$ one has

4.4 $$\begin{aligned} || \, a - E(a | \mathcal {B} ) \, ||_2 \le || a-b ||_2, \ \ \forall \, b \in \mathcal {B} , \text{ with } \text{ equality } \text{ if } \text{ and } \text{ only } \text{ if } b = E(a | \mathcal {B} ), \end{aligned}$$

where $|| \cdot ||_2$ is the $L^2$-norm associated to φ.

A special case of the above: given an $y \in \mathcal {A} ^{\textrm{sa}}$, we will use the notation E(·|y) to refer to the conditional expectation onto the von Neumann subalgebra $\mathcal {B} \subseteq \mathcal {A}$ generated by *y*. This subalgebra can be explicitly described by using functional calculus: $\mathcal {B} = \bigl \{ g(y) \mid g \in \textrm{Bor} _b ( \, \textrm{Spec} (y) \, ) \bigr \},$ where $\textrm{Bor} _b ( \, \textrm{Spec} (y) \, )$ is the space of bounded Borel functions from $\textrm{Spec} (y)$ to $\mathbb {C}$. For $x \in \mathcal {A} ^{\textrm{sa}}$, a convenient way of describing E(x|y) is thus in the form E(x|y)=h(y), where $h: \textrm{Spec} (y) \rightarrow \mathbb {R}$ is a bounded Borel function such that

4.5 $$\begin{aligned} \varphi (x \, g(y))=\varphi \bigl ( \, h(y)\, g(y) \, \bigr ), \ \ \forall \, g \in \textrm{Bor} _b ( \, \textrm{Spec} (y) \, ). \end{aligned}$$

Note that, in view of (4.4), *h*(*y*) is the unique element of the von Neumann algebra generated by *y* which is at minimal distance from *x*, in the $|| \cdot ||_2$-norm associated to φ.

Let us now clarify how the disintegration of the overlap measure appears in calculations of conditional expectations.

### Proposition 4.3

Let $x,y \in \mathcal {A} ^{\textrm{sa}}$ and let $f: \textrm{Spec} (x) \rightarrow \mathbb {R}$ be a bounded Borel function. Consider the element $f(x) \in \mathcal {A} ^{\textrm{sa}}$ obtained by functional calculus, and then consider the conditional expectation $E( f(x) \, | \, y )$. On the other hand, let $\mu ^{( \textrm{ov} )}_{x,y}$ be the overlap measure of *x* and *y*, and consider the disintegration $( k_t^{(2)} )_{t \in \textrm{Spec} (y)}$ of $\mu ^{( \textrm{ov} )}_{x,y}$ that was mentioned in Remark and Notation 4.1. One has that

4.6 $$\begin{aligned} E( f(x) \mid y ) = h(y) \ \text{(functional } \text{ calculus } \text{ of } y), \end{aligned}$$

where $h: \textrm{Spec} (y) \rightarrow \mathbb {R}$ is obtained by fiber-wise integration as in (4.1),

4.7 $$\begin{aligned} h(t) := \int _{ \textrm{Spec} (x)} f (s) \, d k_t^{(2)} (s), \text{ for } t \in \textrm{Spec} (y). \end{aligned}$$

### Proof

Let $\mathcal {B} = \bigl \{ g(y) \mid g \in \textrm{Bor} _b ( \textrm{Spec} (y) ) \bigr \}$ be the von Neumann subalgebra of $\mathcal {A}$ generated by *y*. We have $h(y) \in \mathcal {B}$, and in order to establish (4.6) we need to check that

4.8 $$\begin{aligned} \varphi ( f(x) g(y) ) = \varphi ( h(y) g(y) ), \ \ \forall \, g \in \textrm{Bor} _b ( \textrm{Spec} (y) ). \end{aligned}$$

And indeed, by starting from the left-hand side of (4.8), we can write:

$$\begin{aligned} \varphi ( f(x) g(y) )&= \int _{ \textrm{Spec} (x) \times \textrm{Spec} (y)} f(s) g(t) \, d \mu ^{( \textrm{ov} )}_{x,y} (s,t) \ \text{(by } \text{(3.1)) } \\&= \int _{ \textrm{Spec} (y)} \Bigl [ \, \int _{ \textrm{Spec} (x)} f(s) \, d k_t^{(2)} (s) \, \Bigr ] \, g(t) \, d \mu _y (t) \ \text{(by } \text{(4.2)) } \\&= \int _{ \textrm{Spec} (y)} h(t) g(t) \, d \mu _y (t) \ \text{(by } \text{ how } h \text{ is } \text{ defined) } \\&= \varphi (h(y) g(y)) \ \text{(by } \text{ how } \mu _y \text{ is } \text{ defined). } \end{aligned}$$

The latter quantity is the right-hand side of (4.8), as required. □

### Remark 4.4

In the setting of the preceding proposition, the function $h: \textrm{Spec} (y) \rightarrow \mathbb {C}$ defined in Equation (4.7) has in particular the property that

4.9 $$\begin{aligned} \int _{ \textrm{Spec} (y)} h (t) \, d \mu _y (t) = \int _{ \textrm{Spec} (x)} f(s) \, d \mu _x (s). \end{aligned}$$

Indeed, both sides of (4.9) are found to be equal to $\varphi ( \, f(x) \, )$, where on the left-hand side we start from $\varphi ( \, h(y) \, )$, re-write it as $\varphi ( \, E(f(x) \mid y) \, )$, and use the fact that $\varphi \circ E( \cdot | y ) = \varphi$.

We record here the following consequence of (4.9), which will be used in Section 6 below:

4.10 $$\begin{aligned} \left\{ \begin{array}{l} \text{ Let } V \subseteq \textrm{Spec} (x) \text{ be } \text{ a } \text{ Borel } \text{ set } \text{ with } \mu _x (V) = 1. \\ \text{ There } \text{ exists } \text{ a } \text{ Borel } \text{ set } N \subseteq \textrm{Spec} (y), \text{ with } \mu _y (N) = 0, \\ \text{ such } \text{ that } k_t^{(2)} (V) = 1 \text{ for } \text{ every } t \in \textrm{Spec} (y) \setminus N. \end{array} \right. \end{aligned}$$

For the verification of (4.10) we let $f: \textrm{Spec} (x) \rightarrow \mathbb {R}$ be the indicator function of $\textrm{Spec} (x) \setminus V$, which forces the quantities in (4.9) to be equal to 0, and where the function *h* on the left-hand side has taken the form $h(t) = 1 - k_t^{(2)} (V), \, t \in \textrm{Spec} (y).$ Since *h* is non-negative with $\int h \, d \mu _y = 0$, we get the conclusion stated in (4.10).

## Review of some subordination results in free probability

We will consider several analytic transforms which characterize probability measures on the real line, as follows.

### Notation 5.1

Let μ be a probability measure on the Borel sigma-algebra of $\mathbb {R}$. We will use the customary notation $G_{\mu }$ for the Cauchy transform of μ, defined by

$$\begin{aligned} G_{\mu } (z) := \int _{ \mathbb {R} } \frac{1}{z-t}d\mu (t), \ \ z \in \mathbb {C} \setminus \textrm{supp} ( \mu ) \end{aligned}$$

(where $\textrm{supp} ( \mu ) \subseteq \mathbb {R}$ is the support of μ). Then for $z \in \mathbb {C} \setminus \{ 0 \}$ such that $1/z \not \in \textrm{supp} ( \mu )$ we define

$$\begin{aligned} \psi _{\mu } (z) := \int _{ \mathbb {R} }\frac{zt}{1-zt}d\mu (t) \text{ and } \eta _{\mu } (z) := \frac{\psi _{\mu } (z)}{1+\psi _{\mu } (z)}. \end{aligned}$$

We will refer to the functions $\psi _{\mu }$ and $\eta _{\mu }$ by calling them *moment transform* and respectively *Boolean-cumulant transform* of μ.

We will also make occasional use of the *Hilbert transform* of μ, which is defined as

$$\begin{aligned} H_\mu (t)=\frac{1}{\pi }\lim _{\varepsilon \rightarrow 0^+} Re (G_{\mu }(t+i\varepsilon )), \end{aligned}$$

with the latter limit known to exist for Lebesgue almost every $t \in \mathbb {R}$ (see e.g. [29, Chapter X.3]).

The next lemma records some basic properties of the Cauchy transform.

### Lemma 5.2

(Cf. [5, Lemma 2.17], and the proof references indicated there.)

Let μ be a Borel probability measure on $\mathbb {R}$, and denote by $\mu ^s$, and $\mu ^{ac}$ the singular and absolutely continuous (with respect to Lebesgue measure) parts of μ.

$1^o$ For $\mu ^s$-almost all $x\in \mathbb {R}$, the non-tangential limit of the Cauchy transform $G_\mu$ at *x* is infinite : $\sphericalangle \lim _{z\rightarrow x}G_\mu (z)=\infty$.

$2^o$ For every $x\in \mathbb {R}$, we have $\mu (\{x\})=\sphericalangle \lim _{z\rightarrow x}(z-x) G_\mu (z)$.

$3^o$ Denote by *f* the density of $\mu ^{ac}$ w.r.t. the Lebesgue measure, then for Lebesgue–almost all $x\in \mathbb {R}$, $f(x)=-\frac{1}{\pi }\sphericalangle \lim _{z\rightarrow x} \operatorname {Im} (G_\mu (z))$. □

### Review of subordination results – additive case

Let $( \mathcal {A} ,\varphi )$ be a $W^{*}$-probability space and consider two freely independent selfadjoint elements $a,b \in \mathcal {A}$. We denote the distributions of *a* and *b* with respect to φ by μ and ν, respectively; note that, due to the free independence of *a* and *b*, the distribution of a+b is then the free additive convolution $\mu \boxplus \nu$.

As proved in [13], there exists an analytic map $\omega : \mathbb {C} ^{+} \rightarrow \mathbb {C} ^{+}$, uniquely determined, with the following properties: (i) $G_{\mu \boxplus \nu }(z) = G_{\mu }(\omega (z))$ for all $z \in \mathbb {C} ^{+}$; (ii) ω increases the imaginary part; (iii) $\omega (iy)/(iy) \rightarrow 1 \text{ as } y\rightarrow +\infty .$ This ω is called the *subordination function* of a+b with respect to *a*; if we just want to refer to distributions, ω will be referred to as subordination function of $\mu \boxplus \nu$ with respect to μ.

For the ω considered above, it can be shown that: for every $s \in \mathbb {R}$, the assignment $z \mapsto 1/ ( \omega (z) - s )$ defines a function on $\mathbb {C} ^{+}$ which can be identified as Cauchy transform for a Borel probability measure on $\mathbb {R}$. In the same vein as in the notation from [13, Theorem 3.1], we denote the latter measure as $k_s$; we thus have

5.1 $$\begin{aligned} \int _{ \mathbb {R} }\frac{1}{z-t}dk_s(t)=\frac{1}{\omega (z)-s}, \ \ \forall \, s \in \mathbb {R} \text{ and } z \in \mathbb {C} ^{+}. \end{aligned}$$

Moreover, bundling together the measures $k_s$ turns out to create a Feller-Markov kernel $\mathcal {K}$, where for any bounded Borel function $g: \mathbb {R} \rightarrow \mathbb {C}$ we define the new function $\mathcal {K} g$ by putting

$$ \mathcal {K} g(s)=\int _ \mathbb {R} g(t) dk_s(t), \ \ s \in \mathbb {R} . $$

A relevant point for the considerations of this paper is that the kernel $\mathcal {K}$ can be used for computing conditional expectations onto the $W^{*}$-subalgebra $W^{*} (a) \subseteq \mathcal {A}$ which is generated by *a*; that is, we have

$$ E (g(a+b)|a)= \mathcal {K} g(a), \text{ for } g: \mathbb {R} \rightarrow \mathbb {C} \text{ bounded } \text{ Borel } \text{ function, } $$

where E(·|a) is our notation for conditional expectation onto $W^{*} (a)$.

Finally, a notational comment: it is customary to also consider the subordination function of a+b with respect to *b*. In the few places of the paper where we will do that, we will denote the two subordination functions that arise as $\omega _1$ and $\omega _2$ (hence the ω discussed above will become $\omega _1$). In that setting we have the relations

$$ G_{\mu \boxplus \nu }(z)=G_\mu (\omega _1(z))=G_\nu (\omega _2(z)), \ \ z \in \mathbb {C} ^{+}; $$

another useful formula which is known to hold in connection to $\omega _1$ and $\omega _2$ says that

$$ \omega _1(z)+\omega _2(z)=\frac{1}{G_{\mu \boxplus \nu }(z)} + z, \ \ z\in \mathbb {C} ^+. $$

### Review of subordination results – multiplicative case

Subordination results similar to those reviewed in the preceding subsection also hold true in relation to the operation of free multiplicative convolution on $\mathbb {R} _+=[0, \infty )$. More precisely, let us look once again at a $W^{*}$-probability space $( \mathcal {A} ,\varphi )$, where we now consider two *positive* elements $a,b \in \mathcal {A}$, such that *a* and *b* are freely independent. We denote the distributions of *a* and *b* by μ and ν, respectively, and we consider the free multiplicative convolution $\mu \boxtimes \nu$, which is the distribution of the positive element $a^{1/2} b a^{1/2}$. In connection to this, [13, Theorem 3.6] proves that there exists an analytic map $\omega : \mathbb {C} ^{+} \rightarrow \mathbb {C} ^{+}$, uniquely determined by its properties that it increases the argument of every $z \in \mathbb {C} ^{+}$ and that it satisfies the equation

$$ \psi _{\mu \boxtimes \nu }(z)=\psi _{\mu }(\omega (z)), \ \ z \in \mathbb {C} ^{+}, $$

where the “ψ” notation is used for moment transforms, as in Notation 5.1. This ω is called the *multiplicative subordination function* of $\mu \boxtimes \nu$ with respect to μ.

In the multiplicative setting we are once again interested in a family of Borel probability measures $k_s$, which are now defined on $\mathbb {R} _{+}$, via the following prescription on their moment transforms:

5.2 $$\begin{aligned} \int _{ \mathbb {R} }\frac{zt}{1-zt}dk_s(t) =\frac{\omega (z)s}{1-\omega (z)s}, \ \ z \in \mathbb {C} ^{+}. \end{aligned}$$

The relevance of these measures $k_s$ is that for any bounded Borel function *g* on $\mathbb {R} _+$ we have

$$\begin{aligned} E ( \, g(a^{1/2}ba^{1/2}) \mid a \, ) \ = \ \mathcal {K} g(a), \end{aligned}$$

with the kernel $\mathcal {K}$ defined by the formula

$$\begin{aligned} \mathcal {K} g(s)=\int _{ \mathbb {R} _+} g(t) dk_s(t). \end{aligned}$$

One considers also subordination with respect to ν, if we do so we will use notation $\omega =\omega _1$ and we denote by $\omega _2$ the second subordination function for which we have $\psi _{\mu \boxtimes \nu }=\psi _\nu (\omega _2(z))$. One has $\omega _1(z)\omega _2(z)=z\eta _{\mu \boxtimes \nu }(z)$, where $\eta _{\mu \boxtimes \nu }$ is the Boolean-cumulant transform function mentioned in Notation 5.1.

## Overlap function for x=a and y=a+b, where *a* is free from *b*

In this section, the selfadjoint elements $x,y \in \mathcal {A}$ from the preceding section take the form x=a and y=a+b, where $a,b \in \mathcal {A}$ are selfadjoint and freely independent. We will denote the distributions of *a* and *b* by μ and ν, respectively; due to the freeness assumption on *a* and *b*, the distribution of a+b is then the free additive convolution $\mu \boxplus \nu$. We will show that, in this setting, the overlap measure $\mu ^{( \textrm{ov} )}_{a,a+b}$ is sure to be absolutely continuous with respect to $\mu _a \times \mu _{a+b}$, and we will give an explicit formula for the resulting overlap function, with the important benefit that this will enable us to make explicit computations of conditional expectations of the form E(f(a)|a+b).

### Remark 6.1

By (4.3) and the subordination results reviewed in Subsection 5.1 for any bounded Borel functions *f*, *g* we have

$$\begin{aligned} \varphi (f(a)g(a+b))&=\varphi \left( f(a) E (g(a+b)|a)\right) =\varphi (f(a)\mathcal {K}g(a)) =\int _{ \mathbb {R} } f(s) \mathcal {K}g(s)d\mu (s) \\&=\int _{ \mathbb {R} } f(s) \int _{ \mathbb {R} }g(t)dk_s(t)d\mu (s), \end{aligned}$$

where the probability measures $k_s$ are described by Equation (5.1). Hence for the overlap measure $\mu ^{( \textrm{ov} )}_{a,a+b}$ considered here, the first of the two disintegrations considered in Remark and Notation 4.1 is simply described as

$$ k_s^{(1)}= k_s, \ \ s \in \textrm{Spec} (a). $$

Our goal in this section is that, by starting from the measures $k_s^{(1)}$, we find the second disintegration $k_t^{(2)}$ (with *t* running in $\textrm{Spec} (a+b)$) for $\mu ^{( \textrm{ov} )}_{a,a+b}$. When working towards this goal we will rely on a number of known facts about regularity of free additive convolution and subordination functions, which we review next.

### Remark 6.2

[Regularity of free additive convolution.] Let $\mu ,\nu$ be two Borel probability measures on $\mathbb {R}$, where neither of them is a point mass. We consider the free additive convolution $\mu \boxplus \nu$, and let ω be the subordination function of $\mu \boxplus \nu$ with respect to μ.

$1^o$ $\mu \boxplus \nu$ has at most finitely many atoms $\gamma _i,\, i=1,\ldots ,n$. For every $1 \le i \le n$ there exist $\alpha _i,\beta _i\in \mathbb {R}$, uniquely determined, such that $\alpha _i+\beta _i=\gamma _i$ and $\mu (\{\alpha _i\})+\nu (\{\beta _i\})>1$ (see [11]).

$2^o$ As *z* tends to $\gamma _i$ nontangentially in $\mathbb {C}^+$, the images ω(z) also converge nontangentially to the atom $\alpha _i$ (see [11]).

$3^o$ $\mu \boxplus \nu$ has no singular continuous part (see [5], Theorem 4.1).

$4^o$ Let $(\mu \boxplus \nu )^{ac}$ denote the absolutely continuous part (with respect to Lebesgue measure) of $\mu \boxplus \nu$, and let $f_{\mu \boxplus \nu }$ denote the density of $(\mu \boxplus \nu )^{ac}$ with respect to Lebesgue measure. There exists an open set $U \subset \mathbb {R}$, with $(\mu \boxplus \nu )^{ac} ( \mathbb {R} \setminus U) = 0$, such that $f_{\mu \boxplus \nu }$ is strictly positive on *U* (see [5], Theorem 4.1).

$5^o$ Let *U* be as in $4^o$ above. The subordination function ω can be continued continuously to the real line, and the continuation (still denoted ω) has strictly positive imaginary part at every point x∈U (see [5], Theorem 4.1).

### Proof

Except $5^o$, all statements above are explicitly stated in references. Point $5^o$ requires a bit of explanation. Recall (cf. final paragraph of Section 2.4) that we denote $\omega =\omega _1$, where $\omega _1$ and $\omega _2$ are the subordination functions of $\mu \boxplus \nu$ with respect to μ and to ν, respectively. As stated in the proof of Theorem 4.1 in [5], at least one of the subordination functions $\omega _1, \omega _2$ has strictly positive imaginary part at x∈U. Suppose that the continuation of $\omega _2$ has strictly positive imaginary part at $x\in \mathbb {R}$. We will argue that so does the continuation of $\omega _1=\omega$. One has $\omega _1(z)+\omega _2(z)=1/G_{\mu \boxplus \nu }(z)+z=1/G_\mu (\omega _1(z))+z=1/G_\nu (\omega _2(z))+z$. Then we have

$$\begin{aligned} \omega _1(x)=1/G_\nu (\omega _2(x))-\omega _2(x)+x. \end{aligned}$$

Since for a measure ν not being a point mass we have strict inequality $\operatorname {Im}( 1/G_\nu (z))>\operatorname {Im}(z)$ for $z = \omega _2 (x) \in \mathbb {C} ^{+}$, we see that $\omega _1(x)$ also has strictly positive imaginary part. □

We now proceed with the plan announced above, of examining the measures $k_s$.

### Proposition 6.3

Consider the setting indicated at the beginning of this section (with x=a and y=a+b, where *a*, *b* are freely independent), and let $\mu , \nu$ be the distributions of *a* and *b*, respectively. We assume that neither μ nor ν is a point mass and we consider, in connection to them, the various items of notation used in Review 6.2 above. Let the function $o: \textrm{Spec} (a) \times \textrm{Spec} (a+b) \rightarrow \mathbb {R}_+$ be defined (in $\mu \times (\mu \boxplus \nu )$-almost everywhere sense) by the following formula:

6.1 $$\begin{aligned} \forall s\in \textrm{supp}(\mu ), o(s,t)= {\left\{ \begin{array}{ll} -\frac{1}{\pi } \frac{1}{f_{\mu \boxplus \nu }(t)} \operatorname {Im}\left( \frac{1}{\omega (t)-s}\right) \qquad &  \text{ if } t\in U, \\ \frac{1}{ \mu (\{s\}) } 1_{ \omega (t) = s} &  \text{ if } t \text{ is } \text{ an } \text{ atom } \text{ of } \mu \boxplus \nu , \end{array}\right. } \end{aligned}$$

where $1_{ \omega (t) = s}$ is equal to 1 if ω(t)=s, and equal to 0 otherwise. Consider, on the other hand, the measure $k_s$ from (5.1). Then: for μ-almost every $s \in \mathbb {R}$ we have $k_s\ll \mu \boxplus \nu$ and the corresponding Radon-Nikodym derivative is given by the partial function o(s,·) of the *o* from (6.1).

### Proof

As reviewed in Remark 6.2, there exist a finite set Γ and an open set *U*, with $\Gamma \cap U = \emptyset$, such that: the support of the singular part of $\mu \boxplus \nu$ is Γ, the density $f_{\mu \boxplus \nu }$ of the absolutely continuous part of $\mu \boxplus \nu$ is positive on *U* and $\mu \boxplus \nu (\Gamma \cup U)=1$. These results are obtained by showing that the subordination map ω of $\mu \boxplus \nu$ with respect to μ has a continuous extension $\omega :\mathbb {C}^+ \cup \mathbb {R} \rightarrow \mathbb {C}^+ \cup \mathbb {R} \cup \{\infty \}$, and that $\omega (\Gamma )$ is a set of atoms of μ, while $\omega (U) \subset \mathbb {C}^+$.

Upon invoking Remark 4.4 in connection to the set $V=\Gamma \cup U$ one gets that $k_s(\Gamma \cup U)=1$ for μ-almost all $s\in \mathbb {R}$. For the rest of the proof, let us fix an *s* which has $k_s ( \Gamma \cup U)=1$.

Since the Cauchy transform of $k_s$ takes finite values at all points of *U*, it follows from Lemma 5.2 that:

∙ the singular part of $k_s$ is purely atomic and supported on Γ;

∙ a density of the absolutely continuous part of $k_s$ is $-\pi ^{-1}\operatorname {Im}[(\omega -s)^{-1}] 1_{U}$.

Since the singular part of $\mu \boxplus \nu$ is purely atomic with support Γ and since the density $f_{\mu \boxplus \nu }$ of the absolutely continuous part of $\mu \boxplus \nu$ is positive on *U*, the above bulleted items allow us to conclude that $k_s$ is absolutely continuous with respect to $\mu \boxplus \nu$.

In order to prove that the Radon-Nikodym derivative is indeed given by (6.1), it remains to verify that

6.2 $$\begin{aligned} k_s( \{\gamma \} ) = \frac{\mu \boxplus \nu (\{\gamma \})}{ \mu (\{s\}) } 1_{s=\omega (\gamma )},\quad \gamma \in \Gamma . \end{aligned}$$

Towards this verification, we consider two cases.

– When $s\ne \omega (\gamma )$, (6.2) follows from

$$ k_s(\{\gamma \})=\sphericalangle \lim _{z\rightarrow \gamma }(z-\gamma )G_{k_{s}}(z) =\sphericalangle \lim _{z\rightarrow \gamma } \frac{z-\gamma }{\omega (z)-s}=0. $$

– When $s=\omega (\gamma )$: since ω(z) tends nontangentially to $\omega (\gamma )=s$ as *z* tends nontangentially to γ as shown in [11]), (6.2) follows from taking the nontangential limit as *z* tends to γ in

$$ (z-\gamma )G_{k_{s}}(z)=\frac{z-\gamma }{\omega (z)-s} =\frac{(z-\gamma )G_{\mu \boxplus \nu }(z)}{(\omega (z)-s)G_{\mu }(\omega (z))}. $$

□

### Theorem 6.4

Consider the framework and notation of Proposition 6.3, and let $\mu ^{( \textrm{ov} )}_{a,a+b}$ be the overlap measure for the elements *a* and a+b. One has that

6.3 $$\begin{aligned} d \mu ^{( \textrm{ov} )}_{a,a+b} (s,t) = o(s,t) \, \mu (ds) \ \mu \boxplus \nu (dt), \end{aligned}$$

where the function $o: \textrm{Spec} (a) \times \textrm{Spec} (a+b) \rightarrow \mathbb {R} _{+}$ is defined by Equation (6.1). In particular, this implies that $\mu ^{( \textrm{ov} )}_{a,a+b} \ll \mu \times ( \mu \boxplus \nu )$, and that the corresponding overlap function ${\textrm{o}}_{a,a+b}$ is the function *o* from (6.1).

### Proof

Let ν be the measure on $\textrm{Spec} (a) \times \textrm{Spec} (a+b)$ which is defined by the right-hand side of (6.3), $d \nu (s,t) = o(s,t) \, \mu (ds) \ \mu \boxplus \nu (dt).$ We will verify that ν fulfills the requirement used in order to define the overlap measure $\mu ^{( \textrm{ov} )}_{a,a+b}$.

We know from Proposition 6.3 that, for any given $g\in \textrm{Bor} _b ( \mathbb {R} )$ we have the equality

$$ \int _{ \mathbb {R} } g(t) \, o(s,t) \, d (\mu \boxplus \nu ) (t) =\int _{ \mathbb {R} } g(t)dk_s(t), $$

holding for μ-almost every $s\in \mathbb {R}$. By using Fubini’s Theorem and Remark 6.1, we then see that for every $f,g \in \textrm{Bor} _b ( \mathbb {R} )$ we have

$$\begin{aligned} \int _{ \mathbb {R} ^2} f(s) \, g(t) \, d \nu (s,t)= &   \int _{ \mathbb {R} ^2} f(s) \, g(t) \, o(s,t) \, d\mu (s) \, d ( \mu \boxplus \nu ) (t) \\= &   \int _{ \mathbb {R} }f(s) \left[ \int _{ \mathbb {R} } g(t)\, o(s,t) \, d\mu \boxplus \nu (t)\right] d\mu (s)\\= &   \int _{ \mathbb {R} }f(s) \left[ \int _{ \mathbb {R} } g(t) \, dk_s(t)\right] \, d\mu (s) \\= &   \varphi (f(a)g(a+b)), \end{aligned}$$

as required. □

### Corollary 6.5

Consider the framework and notation from Proposition 6.3 and Theorem 6.4. For every bounded Borel function $f: \textrm{Spec} (a) \rightarrow \mathbb {R}$, one has

$$\begin{aligned} E [ f(a) \mid a +b ] = h(a+b), \end{aligned}$$

where $h: \textrm{Spec} (a+b)\rightarrow \mathbb {C}$ is defined $\mu \boxplus \nu$-almost everywhere by

6.4 $$\begin{aligned} h(t)={\left\{ \begin{array}{ll} -\frac{1}{\pi }\frac{1}{f_{\mu \boxplus \nu }(t)}\operatorname {Im} \left( \int _{ \mathbb {R} }f(s) \frac{1}{\omega (t)-s} d\mu (s)\right) &  \text{ if } t\in U, \\ f(\omega (t))&  \text{ if } t \text{ is } \text{ an } \text{ atom } \text{ of } \mu \boxplus \nu . \end{array}\right. } \end{aligned}$$

### Proof

From Theorem 6.4 and Fubini’s theorem one gets that

$$\begin{aligned} \varphi (f(a)g(a+b))&=\int _{ \mathbb {R} ^2}f(s)g(t) {\textrm{o}}_{a,a+b} (s,t)\mu (ds)\mu \boxplus \nu (dt)\\&=\int _{ \mathbb {R} }\left( \int _{ \mathbb {R} }f(s) {\textrm{o}}_{a,a+b} (s,t)\mu (ds)\right) g(t)\mu \boxplus \nu (dt). \end{aligned}$$

Denoting $h(t)=\int _{ \mathbb {R} }f(s) {\textrm{o}}_{a,a+b} (s,t)\mu (ds)$ we see that

$$\begin{aligned} \varphi (f(a)g(a+b))=\varphi (h(a+b)g(a+b)). \end{aligned}$$

Explicit formula for the function *h* follows directly from the formula (6.1). One should observe that *h* is only defined $(\mu \boxplus \nu )$-almost everywhere, since *U* may be a proper relatively open subset of $\textrm{Spec} (a+b)$. However, this is sufficient for the correct definition of conditional expectation. □

Since the atomic case considered in (6.4) is immediate to apply, we investigate further how the formula (6.4) works in the other case, t∈U. In the next proposition we observe that for f(s)=s one can process the first branch of formula (6.4) a bit further, and evaluate the integral included in it.

### Proposition 6.6

In the particular case where f(s)=s, $s\in \mathbb {R}$, the function *h* defined by (6.4) satisfies

6.5 $$\begin{aligned} h(t) = -\frac{\operatorname {Im}\left( \omega (t) G_{\mu \boxplus \nu }(t)\right) }{\pi f_{\mu \boxplus \nu }(t)}, \ \ \forall \, t\in U. \end{aligned}$$

### Proof

Direct calculation gives

$$\begin{aligned} h(t)&= -\frac{1}{\pi }\int _{ \mathbb {R} } s \frac{1}{f_{\mu \boxplus \nu }(t)} \operatorname {Im} \left( \frac{1}{\omega (t)-s}\right) d\mu (s) \\&= -\frac{1}{\pi f_{\mu \boxplus \nu }(t)} \operatorname {Im}\left( \int _{ \mathbb {R} } \left( \frac{\omega (t)}{\omega (t)-s}-1\right) d\mu (s)\right) \\&= -\frac{\operatorname {Im}\left( \omega (t)G_{\mu }(\omega (t))\right) }{\pi f_{\mu \boxplus \nu }(t)} = -\frac{\operatorname {Im}\left( \omega (t) G_{\mu \boxplus \nu }(t)\right) }{\pi f_{\mu \boxplus \nu }(t)}, \text{ as } \text{ stated. } \end{aligned}$$

□

Formula (6.5) does not seem to further simplify in general, but it allows for some more processing in the case when (the distribution μ of *a* remains general, but) the distribution ν of the element *b* is assumed to be ⊞-infinitely divisible. Under the latter assumption, the *R*-transform $r_\nu$ has an analytic continuation to $\mathbb {C} ^+$ and one can write $\omega (z)=z-r_{\nu }(G_{\mu \boxplus \nu }(z))$. Moreover, if we are in the special situation when *b* is a centred semicircular element of variance $\sigma ^2$ (meaning that ν is the centred semicircle law of variance $\sigma ^2$) then the aforementioned *R*-transform takes the form $r_\nu (z)=\sigma ^2z$, and we find the nice formula which was announced in Section 1.2 of the Introduction under the name of “free additive Tweedie’s formula”. The details of this are given in part $1^o$ of the next example. Part $2^o$ of the example puts into evidence another situation, related to ⊞-convolution powers, when the formula (6.5) takes a particularly nice form.

### Example 6.7

$1^o$ Suppose that *b* is a centred semicircular element with variance $\sigma ^2$. Then $\mu \boxplus \nu$ is absolutely continuous with respect to Lebesgue measure and (as first observed in [12]) the subordination function ω satisfies the equation $\omega (z) = z-\sigma ^2 G_{\mu \boxplus \nu }(z), \ \ z \in \mathbb {C} ^{+},$ which extends by continuity to the case when t∈U. The numerator of the fraction on the right-hand side of (6.5) thus takes the form

$$\begin{aligned} \textrm{Im} \bigl ( \, (t - \sigma ^2 \, G_{\mu \boxplus \nu } (t)) \, G_{\mu \boxplus \nu } (t) \, \bigr )&= t \, \textrm{Im} G_{\mu \boxplus \nu } (t)) - 2 \sigma ^2 \, \textrm{Re} G_{\mu \boxplus \nu } (t) \cdot \textrm{Im} G_{\mu \boxplus \nu } (t) \\&= - \pi t f_{\mu \boxplus \nu } (t) - 2 \sigma ^2 \cdot \pi \, H_{\mu \boxplus \nu } (t) \cdot \bigl ( - \pi \, f_{\mu \boxplus \nu } (t) \bigr ), \end{aligned}$$

where $H_{\mu \boxplus \nu }$ is the Hilbert transform of $\mu \boxplus \nu$, and where at the latter equality sign we took advantage of the fact that the continuous extension of $G_{\mu \boxplus \nu }$ at a point t∈U can be written in the form $G_{\mu \boxplus \nu } (t) = \pi \bigl ( \, H_{\mu \boxplus \nu } (t) - i f_{\mu \boxplus \nu } (t) \, \bigr ).$ Upon plugging the above calculation back into Equation (6.5) we find that

6.6 $$\begin{aligned} h(t) = -\frac{-\pi t f_{\mu \boxplus \nu }(t)+2\pi ^2\sigma ^2 f_{\mu \boxplus \nu }(t) H_{\mu \boxplus \nu }(t)}{\pi f_{\mu \boxplus \nu }(t)} = t - 2\pi \sigma ^2H_{\mu \boxplus \nu }(t), \end{aligned}$$

and this leads to the free additive analogue of Tweedie’s formula that was announced in Equation (1.4) of the Introduction,

$$\begin{aligned} E (a \, | \, a+b) = a+b - 2 \pi \sigma ^2 H_{\mu \boxplus \nu }(a+b). \end{aligned}$$

As already noted in the Introduction, it is remarkable that, in this important special case, the formula for *h* uses solely the distribution of a+b, and does not invoke the (unknown) distribution of the element *a*.

For the record, we mention that in this special case the corresponding overlap function ${\textrm{o}}_{a,a+b}$ is given by the formula

$$ {\textrm{o}}_{a,a+b} (s,t) = \frac{1}{(t-s-\pi \sigma ^2H_{\mu \boxplus \nu }(t))^2 +\pi ^2f_{\mu \boxplus \nu }^2(t)}, \ \text{ for } s\in (-2,2) \text{ and } t\in U. $$

The calculation leading to this formula is similar to the one showed for *h* in (6.6), and is left as exercise to the reader.

$2^o$ If the distributions of *a*, *b* are μ and $\mu ^{\boxplus r}$ respectively for some Borel probability measure μ on $\mathbb {R}$ and some r≥1, then from [7] we know that $G_{\mu ^{\boxplus r+1}}(z)=G_{\mu }(\omega _{r+1}(z))$ with

$$ \omega _{r+1}(z) =\frac{z}{r+1}+\left( 1-\frac{1}{r+1}\right) \, \frac{1}{G_{\mu ^{\boxplus r+1}}(z)}. $$

An immediate application of (6.5) then gives the expected answer,

$$ E (a|a+b)=\frac{a+b}{r+1}. $$

## Multiplicative case

In this section we consider *a*, *b* freely independent positive random variables with respective distributions $\mu , \nu$. Similarly as in the additive case, we first observe that considerations from Section 2.5 imply that the measures $k_s$ defined by

$$\begin{aligned} \int _{ \mathbb {R} }\frac{zt}{1-zt}dk_s(t) =\frac{\omega (z)s}{1-\omega (z)s}, \ \ z \in \mathbb {C} ^{+}. \end{aligned}$$

give us disintegration of the overlap measure $\mu ^{( \textrm{ov} )}_{a,a^{1/2}ba^{1/2}}$ with respect to its first marginal μ. As in the previous section we will show that for μ-almost every *s*, the measure $k_s$ is absolutely continuous with respect to $\mu \boxtimes \nu$. The corresponding Radon-Nikodym derivative will allow us to calculate explicitly conditional expectations of the form $E (f(a) \mid a^{1/2}ba^{1/2})$. We start with a review of regularity of free multiplicative convolution on $\mathbb {R} _+$. In this case we pay special attention to the atoms at 0, which behave differently.

### Remark 7.1

*(Review of regularity of free multiplicative convolution on* $\mathbb {R} _+$ *)* Let $\mu ,\nu$ be two Borel probability measures on $\mathbb {R} _+$ neither of them a point mass; denote by $\mu \boxtimes \nu$ the free multiplicative convolution).

$1^o$ The measure $\mu \boxtimes \nu$ has at most finitely many atoms $\gamma _i,\, i=1,\ldots ,n$. For every $\gamma _i>0$, i=1,…,n there exist uniquely determined $\alpha _i,\beta _i\in (0, \infty )$ such that $\alpha _i\beta _i=\gamma _i$ and $\mu (\{\alpha _i\})+\nu (\{\beta _i\})>1$. Moreover, $\mu \boxtimes \nu (\{0\}) = \max \{ \mu ( \{0\} ), \nu ( \{0\}) \}$ (see [2]).

$2^o$ With notation as in $1^o$ we have as *z* tends to $1/\gamma _i$ nontangentially we have the non-tangential limit $\lim _{z\rightarrow 1/\gamma _i}\omega (z)=1/\alpha _i$, for every i=1,…,n (see [2]).

$3^o$ Measure $\mu \boxtimes \nu$ has no singular continuous part (see [20]).

$4^o$ There exists a closed set $0\in E\subset \mathbb {R} _+$ of Lebesgue measure zero such that the density of $(\mu \boxtimes \nu )^{ac}$, denoted by $f_{\mu \boxtimes \nu }$, is analytic on $\mathbb {R} _{+}\setminus E$ (see [3, 4]).

$5^o$ With notation from $4^o$ the subordination function ω can be continued continuously to the real line, and the continuation, still denoted by ω, has strictly positive imaginary part at every point $x\in \mathbb {R}$ such that $1/x\in \textrm{supp}(\mu \boxtimes \nu )^{ac}\setminus E$ (see [3, 4]).

### Proof

Similarly as before, only $5^o$ requires an explanation. We know that $\omega _1(x)\omega _2(x)=x\eta _{\mu \boxtimes \nu }(x)$, hence we have $\frac{\omega _1(x)}{x}=\frac{\eta _{\nu }(\omega _2(x))}{\omega _2(x)}$, if we know that $\omega _2(x)$ has positive imaginary part, then $\frac{\eta _{\nu }(\omega _2(x))}{\omega _2(x)}$ has positive imaginary part, as $\eta _\mu$ strictly increases argument of $\omega _2(x)$ (see [8]). Thus $\omega _1(x)$ has strictly positive imaginary part. □

In the multiplicative case the behaviour of subordination functions at zero requires a bit more attention. It is explained in detail in [20], let us discuss some elementary results here.

### Lemma 7.2

In the framework and notation of Review 7.1 we have

$$\begin{aligned} \lim _{z\rightarrow -\infty } \omega (z)= {\left\{ \begin{array}{ll} -\infty \qquad &  \text{ if } \mu (\{0\})\ge \nu (\{0\}),\\ \psi _{\mu }^{-1}(\nu (\{0\})-1) &  \text{ if } \mu (\{0\})< \nu (\{0\}). \end{array}\right. } \end{aligned}$$

### Proof

For any probability measure $\mu \ne \delta _0$ on $\mathbb {R} _+$ the function $\psi _\mu :(-\infty ,0)\mapsto (\mu (\{0\})-1,0)$ is a homeomorphism. Therefore $\psi _\mu$ has a continuous inverse $\psi _\mu ^{-1}:(\mu (\{0\})-1,0)\mapsto (-\infty ,0)$. We observe that for $z\in (-\infty ,0)$ function $\omega (z)=\psi _\mu ^{-1}(\psi _{\mu \boxtimes \nu }(z))$ is well defined.

From Lebesgue dominated convergence it follows that

$$ \lim _{z\rightarrow -\infty } \psi _{\mu \boxtimes \nu }(z)=\mu \boxtimes \nu (\{0\})-1=\max \{\mu (\{0\}),\nu (\{0\})\}-1. $$

If $\mu \boxtimes \nu (\{0\})=\mu (\{0\})$ then $\lim _{z\rightarrow -\infty }\psi _\mu ^{-1}(\psi _{\mu \boxtimes \nu }(z))=-\infty$. In the case $\mu \boxtimes \nu (\{0\})=\nu (\{0\})>\mu (\{0\})$ we have $\lim _{z\rightarrow -\infty }\psi _\mu ^{-1}(\psi _{\mu \boxtimes \nu }(z))=\psi _{\mu }^{-1}(\nu (\{0\})-1)$, in this case of course $\nu (\{0\})-1\in (\mu (\{0\})-1,0)$, hence $\psi _{\mu }^{-1}(\nu (\{0\})-1)$ is well defined. □

The next proposition states the precise formula that one has for the overlap function in this case. The proposition does not refer to some freely independent positive elements *a*, *b* in an Borel-ncps – but if such *a*, *b* were in the picture, then the function *o* defined in (7.1) would be the overlap function as $o_{a, a^{1/2} b a^{1/2}}$.

### Proposition 7.3

With notation from Review 7.1 above, consider the function

$o: \textrm{supp}(\mu )\times \textrm{supp}(\mu \boxtimes \nu )\rightarrow \mathbb {R} _+$ defined (in $\mu \times \mu \boxtimes \nu$-almost everywhere sense) by the following formula:

7.1 $$\begin{aligned} o(s,t) := {\left\{ \begin{array}{ll} -\frac{1}{\pi } \frac{1}{tf_{\mu \boxtimes \nu }(t)} \operatorname {Im}\left( \frac{1}{1-\omega (1/t)s}\right) \qquad &  \text{ if } s\in \textrm{supp}(\mu )\\ &  \text{ and } t\in \textrm{supp}(\mu \boxtimes \nu )^{ac}\setminus E,\\ \frac{1}{\mu (\{s\})} 1_{\omega (1/t)=1/s}\qquad &  \text{ if } s\in \textrm{supp}(\mu )\\ &  \text{ and } t>0 \text{ is } \text{ an } \text{ atom } \text{ of } \mu \boxtimes \nu ,\\ \frac{1}{\nu (\{0\})}\frac{1}{1-s \psi _\mu ^{-1}(\nu (\{0\})-1)}1_{\nu (\{0\})>\mu (\{0\})} \qquad &  \text{ if } s\in \textrm{supp}(\mu )\setminus \{0\}, t=0,\\ \frac{1}{\mu \boxtimes \nu (\{0\})}1_{\mu \boxtimes \nu (\{0\})>0} \qquad &  \text{ if } s=t=0, \end{array}\right. } \end{aligned}$$

Consider the measure $k_s$ from (5.2). Then: for μ-almost every $s \in \mathbb {R} _+$ we have $k_s\ll \mu \boxtimes \nu$ and the Radon-Nikodym derivative is given by the function o(s,·) (sending t↦o(s,t)).

### Proof

The proof is very similar to the proof of Proposition 6.3, with the set *U* being replaced by $\textrm{supp}(\mu \boxtimes \nu )^{ac}\setminus E$.

After writing

$$\begin{aligned} G_{k_s}(z)=\frac{1}{z}\frac{1}{1-\omega (1/z)s}, \end{aligned}$$

we apply Stieltjes inversion to get the formula in the case $t\in \textrm{supp}(\mu \boxtimes \nu )^{ac}\setminus E$. Atoms different than 0 are also treated analogously.

Let us calculate the size the atoms at 0 of $k_s$. Of course for any measures $\mu ,\nu$ we have $k_0=\delta _0$, which proves the last point in definition of ${\textrm{o}}_{a,a^{1/2}ba^{1/2}}$.

Consider s>0 we have $\lim _{z\rightarrow \infty }\psi _{k_s}(z)=k_s(\{0\})-1$, on the other hand

$$\begin{aligned} \lim _{z\rightarrow -\infty }\psi _{k_s}(z)=\lim _{z\rightarrow -\infty } \frac{\omega (z)s}{1-\omega (z)s}={\left\{ \begin{array}{ll} -1\qquad &  \text{ if } \mu (\{0\})\ge \nu (\{0\})\\ \frac{s\psi _\mu ^{-1}(\nu (\{0\})-1)}{1-s\psi _\mu ^{-1}(\nu (\{0\})-1)}&  \text{ if } \mu (\{0\})<\nu (\{0\}). \end{array}\right. } \end{aligned}$$

Thus if $\mu (\{0\})<\nu (\{0\})$, then for every s>0 we have $k_s(\{0\})=\frac{s\psi _\mu ^{-1}(\nu (\{0\})-1)}{1-s\psi _\mu ^{-1}(\nu (\{0\})-1)}+1=\frac{1}{1-s\psi _\mu ^{-1}(\nu (\{0\})-1)}$, which justifies second line of the definition of ${\textrm{o}}_{a,a^{1/2}ba^{1/2}}$. □

### Theorem 7.4

Let *a*, *b* be freely independent positive random variables in a Borel-ncps $( \mathcal {A} , \varphi )$. Let μ and respectively ν be the distributions of *a* and *b* with respect to φ, and assume that neither of $\mu , \nu$ is a point mass. Then $d \mu ^{( \textrm{ov} )}_{a,a^{1/2}ba^{1/2}} (s,t)={ {\textrm{o}}_{a,a^{1/2}ba^{1/2}} (s,t)} \mu (ds)\,\mu \boxtimes \nu (dt)$. In particular $\mu ^{( \textrm{ov} )}_{a,a^{1/2}ba^{1/2}} \ll \mu \times \mu \boxtimes \nu$ and ${\textrm{o}}_{a,a^{1/2}ba^{1/2}}$ is the overlap function of *a* and $a^{1/2}ba^{1/2}$.

The proof of this theorem is very similar to the one of Theorem 6.4.

### Corollary 7.5

With the notation of Theorem 7.4, for every $f\in \textrm{Bor}_b ( \textrm{Spec} (a))$ we have

$$\begin{aligned} E [ f(a) \mid a^{1/2}ba^{1/2} ] = h(a^{1/2}ba^{1/2}), \end{aligned}$$

where $h: \textrm{Spec} (a^{1/2}ba^{1/2})\rightarrow \mathbb {C}$ is defined (in $\mu \boxtimes \nu$-almost everywhere sense) by

$$\begin{aligned} h(t)={\left\{ \begin{array}{ll} -\frac{1}{\pi t f_{\mu \boxtimes \nu }(t)}\int _{ \mathbb {R} _+}f(s)\operatorname {Im}\left( \frac{1}{1-\omega (1/t)s}\right) d\mu (s)\qquad &  \text{ if } t\in \textrm{supp}(\mu \boxtimes \nu )^{ac}\setminus E,\\ f\left( \frac{1}{\omega (1/t)}\right) &  \text{ if } t >0 \text{ is } \text{ an } \text{ atom } \text{ of } \mu \boxtimes \nu ,\\ \frac{1}{\nu (\{0\})}\int _{ \mathbb {R} _+}\frac{f(s)}{1-s \psi _\mu ^{-1}(\nu (\{0\})-1)}d\mu (s) &  \text{ if } t=0 \text{ and } \mu (\{0\})<\nu (\{0\}),\\ f(0) &  \text{ if } t=0 \text{ and } \mu (\{0\})\ge \nu (\{0\}). \end{array}\right. } \end{aligned}$$

Here again, the proof is exactly the same as in the additive case.

We will calculate the conditional expectation in the case f(s)=s and then we will further specialize *b* to be a free Poisson element, in which case we will retrieve the well-known Ledoit-Péché estimator of covariance matrix from [21]. The calculations are non-trivial only in two cases: t=0 when $\mu (\{0\})<\nu (\{0\})$ and when $t\in \textrm{supp}(\mu \boxtimes \nu )^{ac}\setminus E$.

### Proposition 7.6

With the notation of Theorem 7.4, one has $$ E (a|a^{1/2}ba^{1/2})=h(a^{1/2}ba^{1/2})$$ where $$h: \textrm{Spec} (a^{1/2}ba^{1/2})\rightarrow \mathbb {C}$$ is defined (in $$\mu \boxtimes \nu $$-almost everywhere sense) by

7.2 $$\begin{aligned} h(t)={\left\{ \begin{array}{ll} -\frac{1}{\pi t f_{\mu \boxtimes \nu }(t)}\operatorname {Im}\left( \frac{1}{\omega (1/t)}\psi _{\mu \boxtimes \nu }\left( \frac{1}{t}\right) \right) \qquad &  \text{ if } t\in \textrm{supp}(\mu \boxtimes \nu )^{ac}\setminus E,\\ \frac{1}{\omega (1/t)} \qquad &  \text{ if } t>0 \text{ is } \text{ an } \text{ atom } \text{ of } \mu \boxtimes \nu ,\\ \frac{\nu (\{0\})-1}{\nu (\{0\})\psi _\mu ^{-1}(\nu (\{0\})-1)} \qquad &  \text{ if } t=0 \text{ and } \mu (\{0\})<\nu (\{0\}),\\ 0 \qquad &  \text{ if } t=0 \text{ and } \mu (\{0\})\ge \nu (\{0\}). \end{array}\right. } \end{aligned}$$

### Proof

Indeed in the case t=0 we get

$$\begin{aligned} h(0)&=\frac{1}{\nu (\{0\})}\int _{ \mathbb {R} _+}\frac{s}{1-s \psi _\mu ^{-1}(\nu (\{0\})-1)}d\mu (s) \\  &=\frac{1}{\nu (\{0\})\psi _\mu ^{-1}(\nu (0)-1)}\int _{ \mathbb {R} _+}\frac{s\psi _\mu ^{-1}(\nu (\{0\})-1)}{1-s \psi _\mu ^{-1}(\nu (\{0\})-1)}d\mu (s)\\&=\frac{\psi _\mu \left( \psi _\mu ^{-1}(\nu (\{0\})-1)\right) }{\nu (\{0\})\psi _\mu ^{-1}(\nu (\{0\})-1)}=\frac{\nu (0)-1}{\nu (\{0\})\psi _\mu ^{-1}(\nu (\{0\})-1)}. \end{aligned}$$

In the case $t\in \textrm{supp}(\mu \boxtimes \nu )^{ac}\setminus E$ we get

$$\begin{aligned} h(t)&=-\frac{1}{\pi t f_{\mu \boxtimes \nu }(t)}\operatorname {Im}\left( \int _{ \mathbb {R} _+}\frac{s}{1-\omega (1/t)s}\right) d\mu (s)\\&=-\frac{1}{\pi t f_{\mu \boxtimes \nu }(t)}\operatorname {Im}\left( \frac{1}{\omega (1/t)}\int _{ \mathbb {R} _+}\frac{\omega (1/t)s}{1-\omega (1/t)s}\right) d\mu (s)\\&=-\frac{1}{\pi t f_{\mu \boxtimes \nu }(t)}\operatorname {Im}\left( \frac{1}{\omega (1/t)}\psi _{\mu \boxtimes \nu }\left( \frac{1}{t}\right) \right) . \end{aligned}$$

□

### Example 7.7

Assume further that *b* is a free Poisson element with parameter λ>0. This means in particular that $\psi _\nu ^{-1}(z)=\tfrac{z}{(\lambda +z)(1+z)}$. We have

$$\begin{aligned} \omega _2(z)=\psi _\nu ^{-1}(\psi _{\mu \boxtimes \nu }(z))=\frac{\psi _{\mu \boxtimes \nu }(z)}{(\lambda +\psi _{\mu \boxtimes \nu }(z))(1+\psi _{\mu \boxtimes \nu }(z))}=\frac{\eta _{\mu \boxtimes \nu }(z)}{\lambda +\psi _{\mu \boxtimes \nu }(z)}. \end{aligned}$$

Taking into account that $\omega _1(z)\omega _2(z)=z\eta _{\mu \boxtimes \nu }(z)$, we see that $\omega (z)=\omega _1(z)=z(\lambda +\psi _{\mu \boxtimes \nu }(z))$. Hence we obtain in the case $t\in \textrm{supp}(\mu \boxtimes \nu )^{ac}\setminus E$ that

$$\begin{aligned} h(t)=-\frac{1}{\pi f_{\mu \boxtimes \nu }(t)}\operatorname {Im}\left( \frac{\psi _{\mu \boxtimes \nu }\left( \frac{1}{t}\right) }{\lambda +\psi _{\mu \boxtimes \nu }\left( \frac{1}{t}\right) }\right) . \end{aligned}$$

Using the relation $\psi _{\mu \boxtimes \nu }(z)=\frac{1}{z}G_{\mu \boxtimes \nu }\left( \frac{1}{z}\right) -1$, straightforward processing of the resulting formula gives

$$\begin{aligned} h(t)=\frac{\lambda t}{|\lambda -1+tG_{\mu \boxtimes \nu }(t)|^2}. \end{aligned}$$

If λ<1 and $\mu (\{0\})<\nu (\{0\})=1-\lambda$ then for t=0 we get

$$ h(0)=\frac{-\lambda }{(1-\lambda )\psi _\mu ^{-1}(-\lambda )}. $$

Remember that $\psi _\mu ^{-1}(-\lambda )=\lim _{z\rightarrow -\infty }\omega (z)=\lim _{z\rightarrow -\infty }z(\lambda +\psi _{\mu \boxtimes \nu }(z))$. One easily verifies that $\psi _{\mu \boxtimes \nu }(z)=\psi _{(\mu \boxtimes \nu )^{ac}}(z)$. Then we have $\lim _{z\rightarrow -\infty }\omega (z)=\lim _{z\rightarrow -\infty }z(\lambda +\psi _{(\mu \boxtimes \nu )^{ac}}(z))=\lim _{z\rightarrow -\infty }G_{(\mu \boxtimes \nu )^{ac}}\left( \frac{1}{z}\right) =G_{(\mu \boxtimes \nu )^{ac}}\left( 0\right)$. Finally we obtain

$$h(0)=-\frac{\lambda }{(1-\lambda )G_{(\mu \boxtimes \nu )^{ac}}\left( 0\right) }. $$

One can also find the overlap function, using $\psi _{\mu \boxtimes \nu }(z)=\frac{1}{z}G_{\mu \boxtimes \nu }\left( \frac{1}{z}\right) -1$ and $\omega (z)=\omega _1(z)=z(\lambda +\psi _{\mu \boxtimes \nu }(z))$ the overlap function from Proposition 7.3 in the case $t\in \textrm{supp}(\mu \boxtimes \nu )\setminus E$ and $s\in \textrm{supp}(\mu )$ simplifies to

$$\begin{aligned} o(s,t)=&-\frac{1}{\pi } \frac{1}{t f_{\mu \boxtimes \nu }(t)} \operatorname {Im}\left( \frac{1}{1-\tfrac{s}{t}(\lambda +t G_{\mu \boxtimes \nu }(t)-1)}\right) \\ =&\frac{st}{(t-s(\lambda -1)-st \pi H_{\mu \boxtimes \nu }(t))^2+\pi ^2 s^2 t^2 f_{\mu \boxtimes \nu ^2}(t)}. \end{aligned}$$

### Example 7.8

*(Free multiplicative Brownian motion)* In the additive case our main example of Tweedie’s formula was related to convolution with semicircular distribution, which is the additive analogue of the Gaussian distribution. It is therefore natural to consider conditional expectation in the multiplicative setting, where the multiplicative noise *b* is given by free multiplicative Brownian motion (for simplicity, at time 1). We denote its distribution by ν.

Free multiplicative convolution with free multiplicative Brownian motion has been studied in detail in [30]. It is shown there that $\mu \boxtimes \nu$ is absolutely continuous with respect to Lebesgue measure, and moreover that if $t\in \textrm{supp}(\mu \boxtimes \nu )$ then

$$\begin{aligned} \omega \left( \frac{1}{t}\right) =\frac{1}{t}\exp \left( -\frac{1}{2}\frac{\eta _{\mu \boxtimes \nu }\left( \frac{1}{t}\right) +1}{\eta _{\mu \boxtimes \nu }\left( \frac{1}{t}\right) -1}\right) . \end{aligned}$$

Using the identity $\eta _{\mu \boxtimes \nu }=\tfrac{\psi _{\mu \boxtimes \nu }}{1+\psi _{\mu \boxtimes \nu }}$ we can write

$$\begin{aligned} \omega \left( \frac{1}{t}\right) =\frac{1}{t}\exp \left( \frac{1}{2}+\psi _{\mu \boxtimes \nu }\left( \frac{1}{t}\right) \right) . \end{aligned}$$

Substituting the above in the absolutely continuous part of the formula (7.2), we obtain

$$\begin{aligned} h(t)=-\frac{1}{\pi f_{\mu \boxtimes \nu }(t)} \textrm{Im}\left[ \psi _{\mu \boxtimes \nu }\left( \frac{1}{t}\right) \exp \left( -\frac{1}{2}-\psi _{\mu \boxtimes \nu }\left( \frac{1}{t}\right) \right) \right] \end{aligned}$$

Hence, the resulting formula depends only on the distribution of $a^{1/2}ba^{1/2}$.

## Relation to conditional freeness

In this section, we relate to conditionally free probability theory the general question of determining the conditional expectation E(x∣y) in the case where: y=P(a,b) is a suitable selfadjoint element defined in terms of two freely independent non-commutative variables *a*, *b* in some $W^{*}$-probability space $(\mathcal {A},\varphi )$, and x=f(a) is a selfadjoint element in the von Neumann algebra generated by *a*. (For concrete examples, *P*(*a*, *b*) could be a selfadjoint polynomial in *a* and *b*; or, in a situation where *a* is known to be positive, it could be that $P(a,b) = a^{1/2} b a^{1/2}$.)

Without much loss of generality, the considerations about E(f(a)∣P(a,b)) which are made throughout this section will also take in the additional assumptions that the function *f* (which is a real-valued bounded Borel function supported on the spectrum of *a*) is non-negative and is normalized by the condition that $\varphi \bigl ( \, f(a) \, \bigr ) = 1$.

### Free denoiser as Radon-Nikodym derivative

#### Remark and Notation 8.1

In the framework that was just described, the conditional expectation E(f(a)∣P(a,b)) is a positive element in the von Neumann algebra generated by *P*(*a*, *b*), and can therefore be written as $h \bigl ( \, P(a,b) \, \bigr )$, where *h* is a bounded Borel supported on the spectrum of *P*(*a*, *b*) and with values in [0,∞). We will refer to *h* by calling it *free denoiser* (of *f*(*a*) with respect to *P*(*a*, *b*)). Our point in the present remark is to observe that the free denoiser *h* can be written as a Radon-Nikodym derivative, in connection to two distributions which appear naturally in the discussion. One of the distributions is just the distribution of *P*(*a*, *b*) with respect to the state φ; we will denote this distribution as $\mu _{P(a,b)}^{\varphi }$. For the second distribution, we consider the new state $\chi : \mathcal {A} \rightarrow \mathbb {C}$ defined by putting

$$\begin{aligned} \chi (c) := \varphi ( \, f(a)c \, \, ) \ \ c \in \mathcal {A} , \end{aligned}$$

and we consider the distribution of *P*(*a*, *b*) in $( \mathcal {A} , \chi )$; the latter distribution will be denoted as $\mu _{P(a,b)}^{\chi }$. The claim we make is that:

$$\begin{aligned} \frac{ d \mu _{P(a,b)}^{\chi } }{ d \mu _{P(a,b)}^{\varphi } } = h \ \ \text{(free } \text{ denoiser), } \end{aligned}$$

i.e. that for any real valued bounded Borel function *g* supported on the spectrum of *P*(*a*, *b*) we have

8.1 $$\begin{aligned} \int _{ \mathbb {R} } g (t) \mu _{P(a,b)}^{\chi } ( dt ) = \int _{ \mathbb {R} } g (t) h(t) \mu _{P(a,b)}^{\varphi } ( dt ). \end{aligned}$$

For the verification of the claim, we start from the left-hand side of (8.1) and we write:

$$\begin{aligned}&\int _{ \mathbb {R} } g (t) \mu _{P(a,b)}^{\chi } ( dt ) \\&\quad = \chi \bigl ( \, g( \, P(a,b) \, \bigr ) \text{(by } \text{ the } \text{ definition } \text{ of } \mu _{P(a,b)}^{\chi }) \\&\quad = \varphi \bigl ( \, f(a) \, g( \, P(a,b) \, \bigr ) \text{(by } \text{ the } \text{ definition } \text{ of } \chi ) \\&\quad = \varphi \Bigl ( \, E \bigl ( f(a) \, g( P(a,b) ) \mid P(a,b) \bigr ) \, \Bigr ) \text{(since } E( \cdot \mid P(a,b)) \text{ is } \varphi \text{-invariant) } \\&\quad = \varphi \Bigl ( \, E \bigl ( f(a) \mid P(a,b) \bigr ) \cdot g (P(a,b)) \, \Bigr ) \text{(conditional } \text{ expectation } \text{ property) } \\&\quad = \varphi \bigl ( \, h( P(a,b)) \cdot g (P(a,b)) \, \bigr ) \text{(by } \text{ the } \text{ definition } \text{ of } h) \\&\quad = \varphi \bigl ( \, (g \cdot h) (P(a,b)) \, \bigr ) \\&\quad = \int _{ \mathbb {R} } g (t) h(t) \mu _{P(a,b)}^{\varphi } ( dt ) \text{(by } \text{ the } \text{ definition } \text{ of } \mu _{P(a,b)}^{\varphi }), \end{aligned}$$

thus arriving to the right-hand side of (8.1).

We next move to connecting the two distributions $\mu _{P(a,b)}^{\varphi }, \mu _{P(a,b)}^{\chi }$ to conditionally free probability theory. Towards that end, we will first review (following [15]) some basic definitions and facts of this theory.

### Review of conditionally free probability theory

#### Definition 8.2

$1^o$ A *two-state non-commutative probability space* is a triple $(\mathcal {A},\varphi ,\chi )$ where $(\mathcal {A},\varphi )$ is a $W^{*}$-probability space and $\chi :\mathcal {A}\rightarrow \mathbb {C}$ is another normal state (unital, positive, weakly continuous linear functional) on $\mathcal {A}$.

$2^o$ Let $(\mathcal {A},\varphi ,\chi )$ be a two-state non-commutative probability space and let *a* be a selfadjoint element of $\mathcal {A}$. Then besides the distribution μ of *a* with respect to φ, one can also consider the distribution ν of *a* in the $W^{*}$-probability space $( \mathcal {A} , \chi )$. The couple $( \mu , \nu )$ of compactly supported probability measures on $\mathbb {R}$ that is obtained in this way will be referred to as *distribution of* *a* *in* $( \mathcal {A} , \varphi , \chi )$.

#### Definition 8.3

0 2.01 .320 .320125 Unital subalgebras $(\mathcal {A}_i)_{i\in I}$ of a unital complex algebra $\mathcal {A}$ are said to be conditionally freely independent in the two-state non-commutative probability space $(\mathcal {A},\varphi ,\chi )$ when they are freely independent in $(\mathcal {A},\varphi )$ and in addition the following condition holds: for all n≥2, $i_1\ne i_2\ne \ldots \ne i_n$ in *I* and $a_1\in \mathcal {A}_{i_1}, \ldots , a_n\in \mathcal {A}_{i_n}$ such that $\varphi (a_1)=\ldots =\varphi (a_n)=0$,

$$\begin{aligned} \chi (a_1\cdots a_n)=\chi (a_1)\cdots \chi (a_n). \end{aligned}$$

Accordingly, non-commutative random variables will be said to be conditionally freely independent in the two-state non-commutative probability space $(\mathcal {A},\varphi ,\chi )$ when the von Neumann subalgebras they generate in $\mathcal {A}$ are so.

### Upgrading freeness to conditional freeness

As originally observed in [15], freely independent unital subalgebras $(\mathcal {A}_i)_{i\in I}$ of a non-commutative probability space $(\mathcal {A},\varphi )$ are conditionally freely independent in the two-state non-commutative probability space given by $(\mathcal {A},\varphi ,\varphi )$. We will use the following slightly more general result.

#### Proposition 8.4

Let $(\mathcal {A},\varphi )$ be a non-commutative probability space and $(\mathcal {A}_i)_{i\in I}$ be freely independent unital subalgebras in $(\mathcal {A},\varphi )$. Fix $i_0\in I$ and a positive element $x \in \mathcal {A}_{i_0}$ such that φ(x)=1. Define another state $\chi :\mathcal {A}\rightarrow \mathbb {C}$ by

$$ \chi (y):=\varphi (x y),\qquad \forall y\in \mathcal {A}. $$

Then the subalgebras $(\mathcal {A}_i)_{i\in I}$ are conditionally freely independent in $(\mathcal {A},\varphi ,\chi )$.

#### Proof

Let n≥2, $i_1\ne i_2\ne \ldots \ne i_n$ in *I* and $a_1\in \mathcal {A}_{i_1}, \ldots , a_n\in \mathcal {A}_{i_n}$ be such that $\varphi (a_1)=\ldots =\varphi (a_n)=0$. We want to show that

8.2 $$\begin{aligned} \chi (a_1\cdots a_n)=\chi (a_1)\cdots \chi (a_n). \end{aligned}$$

Observe that there always exists $k\in \{1,\ldots ,n\}$ such that $i_k\ne i_0$. Then, by free independence of $\mathcal {A}_{i_0}$ and $\mathcal {A}_{i_k}$, $\chi (a_k)=\varphi (xa_k)=\varphi (x)\varphi (a_k)=0$ so that the right-hand side of (8.2) vanishes.

To prove that the left-hand side of (8.2) vanishes, we distinguish two cases: $i_0\ne i_1$ or $i_0=i_1$. On the one hand, if $i_0\ne i_1$, then

$$\begin{aligned} \chi (a_1\cdots a_n)= &   \varphi (xa_1\cdots a_n)\\= &   \varphi ((x-1)a_1\cdots a_n)+\varphi (a_1\cdots a_n)\\= &   0, \end{aligned}$$

where we used twice in the last line free independence of $(\mathcal {A}_i)_{i\in I}$. On the other hand, if $i_0=i_1$, then

$$\begin{aligned} \chi (a_1a_2\cdots a_n)= &   \varphi (xa_1a_2\cdots a_n)\\= &   \varphi ((xa_1-\varphi (xa_1))a_2\cdots a_n)+\varphi (xa_1)\varphi (a_2\cdots a_n)\\= &   0, \end{aligned}$$

where we again used twice in the last line free independence of $(\mathcal {A}_i)_{i\in I}$. In any case, we have proved that

$$\begin{aligned} \chi (a_1\cdots a_n)=0=\chi (a_1)\cdots \chi (a_n), \end{aligned}$$

as required.□

### Conditional expectations and conditional freeness

#### Definition 8.5

Let *a*, *b* be conditionally freely independent selfadjoint non-commutative random variables with respective distributions $(\mu _1,\mu _2)$ and $(\nu _1,\nu _2)$ in some two-state non-commutative probability space $(\mathcal {A},\varphi ,\chi )$ and *P* be a selfadjoint polynomial in two non-commuting variables. We will denote by $(\mu _1\Box ^P\nu _1,(\mu _1,\mu _2)\Box _c^P(\nu _1,\nu _2))$ the distribution of the non-commutative random variable *P*(*a*, *b*) in $(\mathcal {A},\varphi ,\chi )$.

#### Lemma 8.6

Let *a*, *b* be freely independent selfadjoint non-commutative random variables with respective distributions $\mu , \nu$ in a $W^{*}$-probability space $(\mathcal {A},\varphi )$, $f:\mathbb {R}\rightarrow \mathbb {R}$ be a bounded non-negative Borel-measurable function such that φ(f(a))=1 and *P* be a selfadjoint polynomial in two non-commuting variables. Define another state $\chi :\mathcal {A}\rightarrow \mathbb {C}$ by

$$\begin{aligned} \chi (y):=\varphi (f(a)y),\qquad \forall y\in \mathcal {A}. \end{aligned}$$

Then the distribution of the non-commutative random variable *P*(*a*, *b*) in the two-state non-commutative probability space $(\mathcal {A},\varphi ,\chi )$ is $(\mu \Box ^P\nu ,(\mu ,f\cdot \mu )\Box _c^P(\nu ,\nu ))$, where $f \cdot \mu$ denotes the measure absolutely continuous with respect to μ, with Radon–Nikodym derivative equal to *f*.

#### Proof

By assumption, the respective distributions of *a* and *b* with respect to φ are μ and ν. We check then that the respective distributions of *a* and *b* with respect to χ are $f\cdot \mu$ and ν: for any bounded Borel-measurable function $g:\mathbb {R}\rightarrow \mathbb {R}$,

$$\begin{aligned} \chi (g(a))=\varphi (f(a)g(a))=\int _{\mathbb {R}}f(s)g(s)\mu (ds)=\int _{\mathbb {R}}g(s)(f\cdot \mu )(ds); \end{aligned}$$

$$\begin{aligned} \chi (g(b))=\varphi (f(a)g(b))=\varphi (f(a))\varphi (g(b))=\int _{\mathbb {R}}g(s)\nu (ds), \end{aligned}$$

where we have used that *a* and *b* are freely independent and φ(f(a))=1. Now observe that *a* and *b* are conditionally freely independent in $(\mathcal {A},\varphi ,\chi )$ by a direct application of Proposition 8.4 (with x=f(a)) to the von Neumann subalgebras generated by *a* and *b*, and the conclusion follows. □

#### Lemma 8.7

Let $\mu , \nu$ be two compactly supported Borel probability measures on $\mathbb {R}$, $f:\mathbb {R}\rightarrow \mathbb {R}$ be a bounded non-negative Borel-measurable function such that $\int _{\mathbb {R}}f(x)\mu (dx)=1$ and *P* be a selfadjoint polynomial in two non-commuting variables. Then $(\mu ,f\cdot \mu )\Box _c^P(\nu ,\nu )$ is absolutely continuous with respect to $\mu \Box ^P\nu$.

#### Proof

Let *a*, *b* be free such that $a\sim \mu$ and $b\sim \nu$. Let *E* be a Borel subset of $\mathbb {R}$ such that $(\mu \Box ^P\nu )(E)=0$. By the preceding Lemma,

$$\begin{aligned}\left( (\mu ,f\cdot \mu )\Box _c^P(\nu ,\nu )\right) (E)= &   \chi (\textbf{1}_E(P(a,b)))\\= &   \varphi (f(a)\textbf{1}_E(P(a,b)))\\\le &   \sqrt{\varphi (f(a)^2)}\sqrt{\varphi (\textbf{1}_E(P(a,b)))}\\\le &   \Vert f\Vert _{\infty }\sqrt{(\mu \Box ^P\nu )(E)}=0. \end{aligned}$$

This proves absolute continuity of $(\mu ,f\cdot \mu )\Box _c^P(\nu ,\nu )$ with respect to $\mu \Box ^P\nu$. □

The main result of this section is the following.

#### Theorem 8.8

Let *a*, *b* be freely independent selfadjoint non-commutative random variables with respective distributions $\mu , \nu$ in a $W^{*}$-probability space $(\mathcal {A},\varphi )$. Let $f:\mathbb {R}\rightarrow \mathbb {R}$ be a bounded non-negative Borel-measurable function such that φ(f(a))=1, and let *P* be a selfadjoint polynomial in two non-commuting variables. Then

$$ E(f(a)\mid P(a,b)) = \frac{d\left( (\mu ,f\cdot \mu )\Box _c^P(\nu ,\nu )\right) }{d\left( \mu \Box ^P\nu \right) }(P(a,b)). $$

#### Proof

As explained in Remark 8.1, the free denoiser, that is the bounded Borel-measurable function $h:\mathbb {R}\rightarrow \mathbb {R}$ such that

$$\begin{aligned} E(f(a)\mid P(a,b))=h(P(a,b)) \end{aligned}$$

is given by the Radon-Nikodym derivative

$$ h=\frac{ d \mu _{P(a,b)}^{\chi } }{ d \mu _{P(a,b)}^{\varphi } }. $$

Then, according to Lemma 8.6, $(\mu _{P(a,b)}^{\varphi },\mu _{P(a,b)}^{\chi })=(\mu \Box ^P\nu ,(\mu ,f\cdot \mu )\Box _c^P(\nu ,\nu ))$, which concludes the proof. □

#### Remark 8.9

The theorem above extends to any bounded Borel function *f*. Indeed, write

$$ f = f^{+} - f^{-}, $$

where $f^{+}$ and $f^{-}$ denote the positive and negative parts of *f*, respectively. By linearity of conditional expectation,

$$ \mathbb {E}\bigl (f(a)\mid P(a,b)\bigr ) = \mathbb {E}\bigl (f^{+}(a)\mid P(a,b)\bigr ) - \mathbb {E}\bigl (f^{-}(a)\mid P(a,b)\bigr ). $$

Since $f^{+}$ and $f^{-}$ are nonnegative and bounded, the theorem applies to each term separately. In particular, to compute $\mathbb {E}(f^{+}(a)\mid P(a,b))$, we apply the theorem to the normalized function

$$ t \mapsto \frac{f^{+}(t)}{\varphi (f^{+}(a))}, $$

and proceed analogously for $\mathbb {E}(f^{-}(a)\mid P(a,b))$.

#### Example 8.10

In order to illustrate how Theorem 8.8 works, let us work out the didactical example when the distributions $\mu , \nu$ of our freely independent elements *a* and *b* are just given as $\mu =\nu =\frac{1}{2}\delta _{0}+\frac{1}{2}\delta _{2}$, and we want to compute E(a∣a+b). (So, in the setting of Theorem 8.8, the function *f* is a suitable truncation of f(s)=s, and the polynomial *P*(*a*, *b*) is a+b.) We know from the beginning, due to symmetry considerations, that the free denoiser *h* will come out, in this case, as h(t)=t/2; let us, nevertheless, see how c-freeness considerations confirm this result.

We first recall the well-known fact that the distribution $\mu \boxplus \nu$ of a+b is the arcsine distribution, which is absolutely continuous with respect to Lebesgue measure with density $\frac{1}{\pi \sqrt{t(4-t)}}\textbf{1}_{]0;4[}(t)$, and has reciprocal Cauchy transform $F_{\mu \boxplus \nu }(z)=\sqrt{z(z-4)}$.

It is easy to check that the probability distribution $f\cdot \mu$ is the point mass at 2, with reciprocal Cauchy transform $F_{f\cdot \mu }(\omega )=\omega -2$ and that the subordination function is $\omega (z)=\frac{1}{2}z+\frac{1}{2}\sqrt{z(z-4)}$. Hence, using Corollary 4 from [6], the reciprocal Cauchy transform of the c-free convolution $(\mu ,f\cdot \mu )\boxplus _c (\nu ,\nu )$ is given by:

$$\begin{aligned} F_{(\mu ,f\cdot \mu )\boxplus _c (\nu ,\nu )}(z)=F_{f\cdot \mu }(\omega (z))=\frac{1}{2}z+\frac{1}{2}\sqrt{z(z-4)}-2. \end{aligned}$$

By Stieltjes inversion formula, one gets that the density of $(\mu ,f\cdot \mu )\boxplus _c (\nu ,\nu )$ is equal to

$$\begin{aligned} -\frac{1}{\pi }\operatorname {Im}G_{(\mu ,f\cdot \mu )\boxplus _c (\nu ,\nu )}(t)=\frac{\operatorname {Im} F_{(\mu ,f\cdot \mu )\boxplus _c (\nu ,\nu )}(t)}{\pi |F_{(\mu ,f\cdot \mu )\boxplus _c (\nu ,\nu )}(t)|^2}=\frac{\sqrt{t}}{2\pi \sqrt{4-t}}\textbf{1}_{]0;4[}(t). \end{aligned}$$

Finally, the free denoiser is $h(t)=\frac{t}{2}$, as expected.

We note here that, due to the fact that the spectrum of *a* is the set $\{ 0, 2 \}$, we can easily extend the formula discussed above to

$$ E(f(a)\vert a+b)=E \left( \frac{f(2)-f(0)}{2}a+f(0)\vert a+b\right) =\frac{f(2)-f(0)}{2}\frac{a+b}{2}+f(0), $$

holding for a general bounded Borel function $f:\mathbb {R}\rightarrow \mathbb {R}$.

#### Example 8.11

*(Compression with a free projection)* Let us consider *p* a projection with 0<φ(p)<1 and a free, positive element *a* with φ(a)=1. We will calculate *E*(*a*|*pap*), observe that overlap measure technique developed in Section 5 allows for computing *E*(*p*|*pap*) and for *E*(*a*|*pap*) we need to use the technique developed in this section. As we mentioned before the theory of finding distributions of polynomials in c-free variables is not well developed, but in this case we can bypass this difficulty.

Define $\chi (c)=\varphi (a c)$. Of course we have $\mu ^\varphi _{pap}=\mu _a\boxtimes \mu _{p}$. On the other hand χ(1)=1 and for n≥1 we have

$$\begin{aligned} \chi \left( (pap)^n\right) =\varphi \left( (pap)^{n+1}\right) . \end{aligned}$$

This gives immediately

$$\begin{aligned} G_{\mu ^{\chi }_{pap}}(z)=\frac{1-\varphi (p)}{z}+z\left( G_{\mu _a\boxtimes \mu _p}(z)-\frac{1}{z}\right) . \end{aligned}$$

Hence we see that E(a|pap)=h(pap) with

$$\begin{aligned} h(t)= {\left\{ \begin{array}{ll} \frac{1-\varphi (p)}{\mu _a\boxtimes \mu _p(\{0\})} \qquad & \text{ for } t=0,\\ t & \text{ for } t>0. \end{array}\right. } \end{aligned}$$

### Some more details on E(a∣a+b) and $E( a \mid a^{1/2}b a^{1/2})$

In this subsection we use the two-state framework to derive compact formulas for E(a∣a+b) and for $E(a\mid a^{1/2}ba^{1/2})$.

We begin with the additive case. Assume that φ(a)=1. We note that when one is interested only in E(a∣a+b), a formula can be derived directly from Biane’s subordination. Namely, define $\chi (c)=\varphi (ac)$ then

$$\begin{aligned} G^{\chi }_{a+b}(z)&=\varphi \left( \! a\frac{1}{z-a-b}\!\right) =\varphi \left( \! a\frac{1}{\omega (z)-a}\! \right) =\varphi \left( \!\frac{a-\omega (z)}{\omega (z)-a}+\omega (z)\frac{1}{\omega (z)-a}\!\right) \\  &=\omega (z)G_{\mu \boxplus \nu }(z)-1. \end{aligned}$$

Since the conditional expectation is given by the Radon-Nikodym derivative with respect to $\mu \boxplus \nu$ which has only an absolutely continuous part and a discrete part, it follows that

8.3 $$\begin{aligned} h(t)=\lim _{\varepsilon \rightarrow 0^+} \frac{\operatorname {Im}\Bigl (G_{\mu \boxplus \nu }(t+i\varepsilon )\,\omega (t+i\varepsilon )\Bigr )}{\operatorname {Im}\Bigl (G_{\mu \boxplus \nu }(t+i\varepsilon )\Bigr )}. \end{aligned}$$

Indeed, (8.3) clearly holds for t∈U, where *U* is the set on which the density of the absolutely continuous part of $\mu \boxplus \nu$ is positive. For the atomic part, recall that if *t* is an atom of a probability measure σ on $\mathbb {R}$, then $\sigma (\{ t \}) =\lim _{\varepsilon \rightarrow 0^+} i \varepsilon G_{\sigma }( t + i \varepsilon ) =- \lim _{\varepsilon \rightarrow 0^+} \varepsilon \operatorname {Im} G_\sigma ( t + i\varepsilon )$ (cf. Lemma 5.2). Applying this to $\sigma = \mu \boxplus \nu$, we find that if *t* is an atom of $\mu \boxplus \nu$, then

$$ h(t)=\lim _{\varepsilon \rightarrow 0^+}\frac{\varepsilon \operatorname {Im}\Bigl (G_{\mu \boxplus \nu }(t+i\varepsilon )\,\omega (t+i\varepsilon )\Bigr )}{\varepsilon \operatorname {Im}\Bigl (G_{\mu \boxplus \nu }(t+i\varepsilon )\Bigr )}=\lim _{\varepsilon \rightarrow 0^+}\frac{\operatorname {Im}\Bigl (G_{\mu \boxplus \nu }(t+i\varepsilon )\,\omega (t+i\varepsilon )\Bigr )}{\operatorname {Im}\Bigl (G_{\mu \boxplus \nu }(t+i\varepsilon )\Bigr )}. $$

It is instructive to verify that, at an atom *t* of $\mu \boxplus \nu$, (8.3) yields the same value *h*(*t*) as we had in Proposition 1.3. Indeed, let us consider such a *t*, which we write as $t = \alpha +\beta$ with $\mu (\{\alpha \})+\nu (\{\beta \})>1$. We then have

$$\begin{aligned} h(t)&=\lim _{\varepsilon \rightarrow 0^+}\frac{\operatorname {Im}\Bigl (G_{\mu \boxplus \nu }(t+i\varepsilon )\,\omega (t+i\varepsilon )\Bigr )}{\operatorname {Im}\Bigl (G_{\mu \boxplus \nu }(t+i\varepsilon )\Bigr )}\\&=\alpha +\lim _{\varepsilon \rightarrow 0^+}\frac{\operatorname {Im}\Bigl (G_{\mu \boxplus \nu }(t+i\varepsilon )\,\left( \omega (t+i\varepsilon )-\alpha \right) \Bigr )}{\operatorname {Im}\Bigl (G_{\mu \boxplus \nu }(t+i\varepsilon )\Bigr )}\\&=\alpha +\lim _{\varepsilon \rightarrow 0^+}\frac{\operatorname {Im}\Bigl (G_{\mu }(\omega (t+i\varepsilon ))\,\left( \omega (t+i\varepsilon )-\alpha \right) \Bigr )}{\operatorname {Im}\Bigl (G_{\mu \boxplus \nu }(t+i\varepsilon )\Bigr )}. \end{aligned}$$

But, similarly to what we discussed in the proof of Proposition 6.3, we have that

$$ \lim _{\varepsilon \rightarrow 0^+} \operatorname {Im}\Bigl (G_{\mu }(\omega (t+i\varepsilon ))\,\left( \omega (t+i\varepsilon )-\alpha \right) \Bigr )=\mu (\{\alpha \}), $$

while ${\operatorname {Im}\Bigl (G_{\mu \boxplus \nu }(t+i\varepsilon )\Bigr )}$ diverges; this implies, altogether, that $h(t)=\alpha =\omega (t)$.

Similar considerations to those shown above apply to the multiplicative case. When we are interested in $E(a|a^{1/2}ba^{1/2})$ (with χ defined as above) we have

$$\begin{aligned} 1+\psi ^{\chi }_{a^{1/2}ba^{1/2}}(z)=\varphi (a(1-za^{1/2}ba^{1/2})^{-1})=\varphi (a(1-\omega (z)a)^{-1})=\frac{\psi ^{\varphi }_{a^{1/2}ba^{1/2}}(z)}{\omega (z)}. \end{aligned}$$

Hence,

$$\begin{aligned} G^{\chi }_{a^{1/2}ba^{1/2}}(z)=\frac{G_{a^{1/2}ba^{1/2}}^{\varphi }(z)-\frac{1}{z}}{\omega (\frac{1}{z})}. \end{aligned}$$

Thus for $\mu \boxtimes \nu$ a.e. *t*, we define the free denoiser in the multiplicative case as

$$ h(t)=\lim _{\varepsilon \rightarrow 0^+}\frac{\operatorname {Im}\left( \frac{G_{a^{1/2}ba^{1/2}}^{\varphi }(t+i\varepsilon )-\frac{1}{t+i\varepsilon }}{\omega (\frac{1}{t+i\varepsilon })}\right) }{\operatorname {Im}\left( G_{a^{1/2}ba^{1/2}}^{\varphi }(t+i\varepsilon )\right) }. $$

A simple calculation, similar to the additive case shows that this expression agrees with the one given in (7.2). One point to explain is that the limit $\lim _{\varepsilon \rightarrow 0^+}\omega \left( \frac{1}{i\varepsilon }\right)$ agrees with the corresponding limit at -∞ found in Lemma 7.2; we refer to [20], where this was shown.

## Relation to matrix denoising

As mentioned in the Introduction, the explicit formulas of free denoisers noticed in (1.4) and (1.12) of this paper had previously appeared in [16, 21], derived via random matrix techniques. We will now see that this is not coincidental, as the matrix denoising problem addressed in the aforementioned references relates to free denoising via the phenomenon of asymptotic freeness.

### Weak continuity of overlap measure

In this subsection we record, in the next Proposition 9.1 and its Corollary 9.3, a general observation about the behaviour of overlap measures under convergence in moments for the pairs of selfadjoint elements that are considered.

#### Proposition 9.1

Suppose we are given the following data.

→ $( \mathcal {A} , \varphi )$ is a $W^{*}$-probability space as considered in Example 2.7 and (*x*, *y*) is a pair

     of elements in $\mathcal {A} ^{\textrm{sa}}$, with overlap measure $\mu ^{( \textrm{ov} )}_{x,y}$.

→ For every $N \in \mathbb {N}$, $( \mathcal {A} _N, \varphi _N )$ is a Borel-ncps, from Definition 2.3, and $(x_N,y_N)$

     is a pair of elements in $\mathcal {A} _N^{\textrm{sa}}$, with overlap measure $\mu _{(x_N, y_N)}^{\textrm{ov}}$.

Suppose, moreover, that

9.1 $$\begin{aligned} \lim _{N \rightarrow \infty } \varphi _N \bigl ( x_N^p \, y_N^q \bigr ) = \varphi ( x^p \, y^q ), \ \ \forall \, p,q \in \mathbb {N} \cup \{ 0 \}. \end{aligned}$$

Then it follows that

9.2 $$\begin{aligned} \lim _{N \rightarrow \infty } \int _{ \mathbb {R} ^2} f(s) \, g(t) \, d \mu _{(x_N, y_N)}^{\textrm{ov}} (s,t) = \int _{ \mathbb {R} ^2} f(s) \, g(t) \, d \mu ^{( \textrm{ov} )}_{x,y} (s,t), \ \ \forall \, f,g \in C_{\textrm{pol}} ( \mathbb {R} ), \end{aligned}$$

where $C_{\textrm{pol}} ( \mathbb {R} ) = \{ f \in \textrm{Bor}_{\textrm{pol}} ( \mathbb {R} ) \mid f \text{ is } \text{ continuous } \}$ (the space of continuous functions with polynomial growth from $\mathbb {R}$ to $\mathbb {C}$). □

#### Remark 9.2

$1^o$ The proof of Proposition 9.1 is an application of the well-known “method of moments”, where we start with Equation (9.1) written in the form

$$ \lim _{N \rightarrow \infty } \int _{ \mathbb {R} ^2} s^p \, t^q \, d \mu _{(x_N, y_N)}^{\textrm{ov}} (s,t) = \int _{ \mathbb {R} ^2} s^p \, t^q \, d \mu ^{( \textrm{ov} )}_{x,y} (s,t), \ \ \forall \, p,q \in \mathbb {N} \cup \{ 0 \}, $$

and where an intermediate step of the proof is to infer (by using a 2-dimensional version of Helly’s selection theorem) that the measures $\mu _{(x_N, y_N)}^{\mathrm {(ov)}}$ converge weakly to $\mu ^{( \textrm{ov} )}_{x,y}$. For the said intermediate step it is relevant to note that the measure $\mu ^{( \textrm{ov} )}_{x,y}$, having compact support, is uniquely determined by its moments. The details of this proof are left to the reader.

$2^o$ In Proposition 9.1 we assumed that *x*, *y* live in a $W^{*}$-probability space, while on the $x_N, y_N$ we only made the looser assumption that they live in a Borel-ncps (which could be, in particular, a space of N×N random matrices). As reviewed in Section 4, the $W^{*}$-framework allows us to consider the non-commutative conditional expectation $E( x \, | \, y )$. While the analogous non-commutative conditional expectations may not be guaranteed for the pairs $(x_N, y_N)$, one can nevertheless observe the following immediate consequence of Proposition 9.1, which only refers to $E(x \, | \, y )$.

#### Corollary 9.3

In the framework and notation of Proposition 9.1, one has

9.3 $$\begin{aligned} \lim _{N \rightarrow \infty } || \, x_N - g (y_N) \, ||_{2, \varphi _N} = || \, x - g(y) ||_{2, \varphi } \ge || \, x - E(x \, | \, y ) \, ||_{2, \varphi }, \ \ \forall \, g \in C_{\textrm{pol}} ( \mathbb {R} ), \end{aligned}$$

where $|| \cdot ||_{2, \varphi }$ and $|| \cdot ||_{2, \varphi _N}$ are the $L^2$-norms associated to the spaces $( \mathcal {A} , \varphi )$ and $( \mathcal {A} _N, \varphi _N )$, respectively.

Equation (9.3) has the following meaning: suppose that the denoiser of *x* with respect to *y* is given by a continuous function $h \in C_{\textrm{pol}} ( \mathbb {R} )$ (that is, *h* is such that $E( x \, | \, y ) = h(y)$). Then the same *h* must act as an asymptotic denoiser for $x_N$ with respect to $y_N$, at least in the weak sense that no $g \in C_{\textrm{pol}} ( \mathbb {R} )$ can systematically outperform *h* in the large *N* limit.

### Additive matrix denoising

A rich source of asymptotically free pairs of sequences of selfadjoint non-commutative random variables is provided by random matrices. In this context, matrix denoising is a general problem arising in high-dimensional statistics: an unknown signal represented by a Hermitian N×N random matrix $A_N$ is corrupted by a noise which is itself a Hermitian N×N random matrix $B_N$ and one would like to “recover the signal” $A_N$ as a function of the noisy observation $A_N + B_N$.

In the lineage of [21], Allez, Bouchaud, Bun and Potters addressed in [16] a general problem of additive/multiplicative matrix denoising: given two independent symmetric (respectively semi-positive definite) N×N random matrices $A_N, B_N$, where the distribution of the noise $B_N$ is invariant under the action by conjugation of the orthogonal group, the problem is to find the optimal (in the $L^2$ sense) Rotationally Invariant Estimator (RIE for short) of the signal $A_N$, based on the observation of $C_N=A_N+B_N$ (or $C_N=A_N^{1/2}B_NA_N^{1/2}$, respectively). The optimal RIE is a bounded measurable function $\delta _N:\mathbb {R}\rightarrow \mathbb {R}$ which minimizes $\mathbb {E}[N^{-1}{\text {Tr}}\left( (A_N-\delta _N(C_N))^2\right) ]$. The paper [16] provides arguments supporting the claim that the sequence $(\delta _N)_{N\in \mathbb {N}}$ converges to a function $\delta :\mathbb {R}\rightarrow \mathbb {R}$ which may be expressed by using tools from free probability theory.

When particularized to a N×N GOE-distributed additive noise $B_N$ with variance parameter $\sigma ^2N^{-1}$, the sequence $(\delta _N)_{N\in \mathbb {N}}$ of optimal RIE of $A_N$ in the general additive matrix denoising problem converges to the function $\delta :\mathbb {R}\rightarrow \mathbb {R}$ defined by:

$$ \delta (t)=t-2\sigma ^2\pi H(t), $$

where *H* is the Hilbert transform of the limiting empirical spectral distribution of $A_N+B_N$.

This result may be interpreted in terms of free denoising. Moreover, using this interpretation, we prove the following proposition, stating that no $g \in C_{\textrm{pol}} ( \mathbb {R} )$ can systematically outperform δ as an asymptotic denoiser in the large *N* limit.

#### Proposition 9.4

Let, for each $N\in \mathbb {N}$, $A_N$ be a N×N deterministic symmetric matrix and $B_N$ be a N×N random matrix distributed according to GOE with variance $\sigma ^2N^{-1}$. Assume that

$$ N^{-1}{\text {Tr}}(A_N^k)\underset{N\rightarrow \infty }{\longrightarrow } \int _{\mathbb {R}}x^k\mu (dx), $$

for some compactly supported Borel probability measure μ on $\mathbb {R}$. Let $C_N=A_N+B_N$. Then, for all $g \in C_{\textrm{pol}} ( \mathbb {R} )$,

$$ \lim _{N\rightarrow \infty }\mathbb {E}[N^{-1}{\text {Tr}}\left( (A_N-g(C_N))^2\right) ] \ge \lim _{N\rightarrow \infty }\mathbb {E}[N^{-1}{\text {Tr}}\left( (A_N-\delta (C_N))^2\right) ]. $$

#### Proof

We apply Corollary 9.3 to $x_N=A_N$ and $y_N=C_N$. More precisely, we work in $(\mathcal {A}_N=M_N(L^{\infty -}(\Omega ,\mathcal {F},\mathbb {P})), \varphi _N=\mathbb {E}N^{-1}{\text {Tr}})$, which is a Borel-ncps (see Example 2.9) and observe that $(x_N,y_N)$ is a pair of elements in $\mathcal {A}_N^{\textrm{sa}}$. It is well-known that $(x_N,y_N)_{N\in \mathbb {N}}$ are asymptotically free: in particular, condition (9.1) is satisfied for some pair (x=a,y=a+b) of elements built from freely independent selfadjoint elements *a*, *b* in a $W^{*}$-probability space $(\mathcal {A},\varphi )$. Here, by assumption, *a* is distributed according to μ and, by Wigner’s Theorem, *b* is distributed according to Wigner semicircle law with variance $\sigma ^2$, which is denoted by ν. Corollary 9.3 then implies that, for all $g \in C_{\textrm{pol}} ( \mathbb {R} )$,

$$\begin{aligned} \lim _{N\rightarrow \infty } \mathbb {E}[N^{-1} {\text {Tr}} \left( (A_N-g(C_N))^2\right) ]&= \lim _{N \rightarrow \infty }|| \, x_N - g(y_N) \, ||_{2, \varphi _N}^2 \\&= || \, x - g(y) \, ||_{2, \varphi }^2 \ge || \, x - E(x \, | \, y ) \, ||_{2, \varphi }^2. \end{aligned}$$

It follows from Example 6.7 that $E(x\vert y)=y-2\sigma ^2\pi H_{\mu \boxplus \nu }(y)$, which is precisely δ(y) for the function $\delta :\mathbb {R}\rightarrow \mathbb {R}$ predicted in [16]. Since the Hilbert transform $H_{\mu \boxplus \nu }$ of the free additive convolution $\mu \boxplus \nu$ is continuous (see [12]), the function $\delta :\mathbb {R}\rightarrow \mathbb {R}$ is itself in $C_{\textrm{pol}} ( \mathbb {R} )$ and

$$\begin{aligned} || \, x - E(x \, | \, y ) \, ||_{2, \varphi }^2&=|| \, x - \delta (y) \, ||_{2, \varphi }^2 =\lim _{N \rightarrow \infty }|| \, x_N - \delta (y_N) \, ||_{2, \varphi _N}^2 \\&=\lim _{N\rightarrow \infty }\mathbb {E}[N^{-1}{\text {Tr}}\left( (A_N-\delta (C_N))^2\right) ]. \\ \end{aligned}$$

□

Similarly, when the multiplicative noise $B_N$ is Wishart-distributed with aspect ratio converging towards $\gamma \in (0, \infty )$, [16] recovers the estimator previously derived by Ledoit and Péché in [21] that is reviewed in the following subsection.

### Covariance matrix estimation

Another important instance of the general matrix denoising problem is the so-called covariance matrix estimation. Let $X_1,\ldots ,X_p$ be independent identically distributed random vectors in $\mathbb {C}^N$ with independent standardized entries, and let $\Sigma _N$ be a positive semi-definite Hermitian N×N matrix. The goal is to estimate $\Sigma _N$ from the observation of the independent identically distributed random vectors $\Sigma _N^{1/2}X_1,\ldots ,\Sigma _N^{1/2}X_p$ with common covariance matrix $\Sigma _N$. It is well-known that the sample covariance matrix

$$ Y_N=p^{-1}\sum _{i=1}^p(\Sigma _N^{1/2}X_i)(\Sigma _N^{1/2}X_i)^* $$

is a consistent estimator of $\Sigma _N$ in the regime where *N* is fixed and *p* is large. But in the regime where *p* and *N* are both large with ratio *p*/*N* converging to $\gamma \in (0,\infty )$, the sample covariance matrix $Y_N$ becomes a poor estimator of $\Sigma _N$: it is empirically observed that the eigenvalues of the sample covariance matrix $Y_N$ spread out significantly compared to the eigenvalues of the true covariance matrix $\Sigma _N$. Based on this observation, Ledoit and Péché suggested in [21] to design estimators of $\Sigma _N$ which shrink eigenvalues of $Y_N$ without modifying the corresponding eigenvectors, hence are of the form $\delta (Y_N)$, where $\delta :\mathbb {R}\rightarrow \mathbb {R}$ is a bounded measurable function. At fixed *N*, there is an optimal (in the $L^2$ sense) bounded measurable function $\delta _N:\mathbb {R}\rightarrow \mathbb {R}$.

Assuming that the 12th moment of entries of $X_1,\ldots ,X_p$ is bounded by some constant independent of *N* and *p*, that $\Sigma _N$ is positive definite, with empirical spectral distribution weakly converging to some probability measure μ with compact support included in (0,∞), and assuming that $\gamma \ne 1$, Ledoit and Péché proved that the sequence $(\delta _N)_{N\in \mathbb {N}}$ converges to the function $\delta :\mathbb {R}\rightarrow \mathbb {R}$ defined by:

$$\begin{aligned} \delta (t)=\left\{ \begin{array}{c} \frac{t}{|1-\gamma ^{-1}+\gamma ^{-1}tG(t)|^2},\quad \text{ if } t>0\\ -\frac{\gamma }{(1-\gamma )\underline{G}(0)}\textbf{1}_{\gamma <1},\quad \text{ if } t=0,\\ \end{array} \right. \end{aligned}$$

where *G* is the Stieltjes transform of the limiting empirical spectral distribution of $Y_N$ and $\underline{G}(t)=(1-\gamma ^{-1})t^{-1}+\gamma ^{-1}G(t)$.

This result also has an interpretation in terms of free denoising: one may argue as in the proof of Proposition 9.4 that $(x_N,y_N)=(\Sigma _N,Y_N)$ defines for each $N\in \mathbb {N}$ a pair of selfadjoint elements in a Borel-ncps in such a way that condition (9.1) is satisfied for some pair $(x,y)=(a,a^{1/2}ba^{1/2})$ of elements built from freely independent positive elements *a*, *b* in a $W^*$-probability space. Here, *a* is distributed according to μ and *b* is distributed according to a dilation of the free Poisson distribution with parameter γ. The function δ found in [21] and reviewed above coincides, up to a dilation factor, to the free denoiser computed in Example 7.7; one may then use Corollary 9.3 to conclude that no $g \in C_{\textrm{pol}} ( \mathbb {R} )$ can outperform δ as an asymptotic denoiser in the large *N* limit.

## Data Availability

No datasets were generated or analysed during the current study.

## Appendix A. Multiplicative convolution on the unit circle

Unitary operators do not fit into the framework of this paper, where we consider selfadjoint operators. However we point out that a similar development to the one from Sections 3, 4 and 5 can be done in the case of multiplication of freely independent unitary random variables. For the purpose of this section we fix *u*, *v* freely independent unitary random variables in a $W^*$-probability space, with respective distributions μ and ν. The distribution of *uv* is the free multiplicative convolution of μ and ν, also denoted by $\mu \boxtimes \nu$. One can also define the overlap measure $\mu ^{(ov)}_{u,uv}$, but in this case this measure is defined on the Borel sigma-algebra of $\mathbb {T}^2$.

In [13] Biane proved analogous subordination results in the case of multiplicative convolution on the unit circle. More precisely, for |z|<1 we have :

$$ E\left( \frac{z u v}{1-zuv}\Big |u\right) =\frac{\omega (z)u}{1-\omega (z)u}. $$

Similarly as before, for any $s\in \mathbb {T}$, the formula

$$\psi _{k_s}(z)=\frac{\omega (z) s}{1-\omega (z)s},\quad |z|<1, $$

defines the moment transform of a probability measure $k_s$ supported on $\mathbb {T}$, that is

$$\begin{aligned} \psi _{k_s}(z)=\int _{\mathbb {T}}\frac{z t}{1-zt}k_s(dt). \end{aligned}$$

One can retrieve the measure $k_s$ as a weak limit via

$$\begin{aligned} 2 \pi d k_s(t)=\lim _{r\uparrow 1}( 2 Re(\psi _{k_s}(r \overline{t}))+1 )dt, \end{aligned}$$

where *dt* is the Lebesgue measure on $\mathbb {T}$.

For any bounded Borel function *g* on $\mathbb {T}$ we have

$$\begin{aligned} E(g(uv)|u)= \mathcal {K} g(u) \end{aligned}$$

where kernel $\mathcal {K}$ is defined as

$$\begin{aligned} \mathcal {K} g(s)=\int _{\mathbb {T}} g(t)k_s(dt). \end{aligned}$$

As in the additive and multiplicative case on $\mathbb {R} _+$, Biane’s subordination gives the first disintegration of $\mu ^{(ov)}_{u,uv}$, namely we have

$$\begin{aligned} \varphi (f(u) g(uv))&=\varphi (f(u)E(g(uv)|u))=\varphi (f(u) \mathcal {K} g(u))=\int _{\mathbb {T}} f(s) \mathcal {K} g(s) d\mu (s)\\&=\int _{\mathbb {T}}f(s)\int _{\mathbb {T}}g(t) dk_s(t) d\mu (s). \end{aligned}$$

Regularity and atoms of free multiplicative convolution on the unit circle together with boundary behaviour of subordination functions are well understood (see [2, 4]). Recently in [9] the authors showed that the distribution of *uv* has no singular continuous part. Using similar methods as in Sections 4 and 5, one can show that for μ-almost every $s\in \mathbb {T}$, the measure $k_s$ is absolutely continuous with respect to $\mu \boxtimes \nu$. We have the following.

### Proposition A.1

With notation as above suppose that μ and ν are not point masses. There exists a subset $U\subset \mathbb {T}$ of full measure with respect to $(\mu \boxtimes \nu )^{ac}$ such that for μ-almost every $s \in \mathbb {T}$, $k_s(dt)=o(s,t)\mu \boxtimes \nu (dt)$, where $o:\textrm{supp}(\mu )\times \textrm{supp}(\mu \boxtimes \nu )\rightarrow \mathbb {R}_+$ is defined (in $\mu \times \mu \boxtimes \nu$-almost everywhere sense) by the following formula:

$$\begin{aligned} \forall s\in \textrm{supp}(\mu ), o(s,t)={\left\{ \begin{array}{ll} \frac{1}{2\pi f_{\mu \boxtimes \nu }(t)} Re\left( 2\frac{\omega \left( \overline{t}\right) s}{1-\omega \left( \overline{t}\right) s}+1\right) \qquad & \text{ if } t\in U,\\ \frac{1}{\mu (s)}1_{\omega (\overline{t})=\overline{s}}& \text{ if } t \text{ is } \text{ an } \text{ atom } \text{ of } \mu \boxtimes \nu . \end{array}\right. } \end{aligned}$$

### Theorem A.2

Let *u*, *v* be freely independent unitary random variables in a $W^*$-probability space, with respective distributions μ and ν not being point masses. Then $d\mu ^{(ov)}_{u,uv}(s,t)=o(s,t) \mu (ds)\,\mu \boxtimes \nu (dt)$, where *o* is defined in the previous proposition.

### Corollary A.3

The result above implies that for every $f\in \textrm{Bor}_b ( \mathbb {T} )$ we have

$$\begin{aligned} E [ f(u) \mid uv ] = h(uv), \end{aligned}$$

where $h: \textrm{Spec} (uv)\rightarrow \mathbb {C}$ is defined $\mu \boxtimes \nu$-almost everywhere by

$$\begin{aligned} h(t)={\left\{ \begin{array}{ll} \frac{1}{2 \pi f_{\mu \boxtimes \nu }(t)}\int _{\mathbb {T}}f(s)Re\left( 2\frac{\omega \left( \overline{t}\right) s}{1-\omega \left( \overline{t}\right) s}+1\right) d\mu (s)\qquad &  \text{ if } t\in U,\\ f\left( \overline{\omega (\overline{t})}\right) &  \text{ if } t \text{ is } \text{ an } \text{ atom } \text{ of } \mu \boxtimes \nu . \end{array}\right. } \end{aligned}$$

## References

[1] Arizmendi, O., Tarrago, P., Vargas, C.: Subordination methods for free deconvolution. Ann. Inst. Henri Poincaré Probab. Stat. **56**(4), 2565–2594 (2020)

[2] Belinschi, S.T.: The atoms of the free multiplicative convolution of two probability distributions. Integral Equations Operator Theory **46**(4), 377–386 (2003)

[3] Belinschi, S.T.: Complex analysis methods in non-commutative probability. Thesis (Ph.D.) Indiana University, 102 pages. ProQuest LLC, Ann Arbor, 2005. Also available as arXiv:math/0602343

[4] Belinschi, S.T.: A note on regularity for free convolutions. Ann. Inst. H. Poincaré Probab. Statist. **42**(5), 635–648 (2006)

[5] Belinschi, S.T.: The Lebesgue decomposition of the free additive convolution of two probability distributions. Probab. Theory Related Fields **142**(1–2), 125–150 (2008)

[6] Belinschi, S.T.: C-free convolution for measures with unbounded support Theta Ser. Adv. Math. **10**, (2008)

[7] Belinschi, S.T., Bercovici, H.: Atoms and regularity for measures in a partially defined free convolution semigroup. Math. Z. **248**(4), 665–674 (2004)

[8] Belinschi, S.T., Bercovici, H.: Partially defined semigroups relative to multiplicative free convolution. Int. Math. Res. Not. **2**, 65–101 (2005)

[9] Belinschi, S.T., Bercovici, H., Ho, C.-W.: Regularity for free multiplicative convolution on the unit circle. Pacific J. Math. **322**(2), 243–250 (2023)

[10] Belinschi, S.T., Mai, T., Speicher, R.: Analytic subordination theory of operator-valued free additive convolution and the solution of a general random matrix problem. J. Reine Angew. Math. **732**, 21–53 (2017)

[11] Bercovici, H., Voiculescu, D.: Regularity questions for free convolution. In “Nonself-adjoint operator algebras, operator theory, and related topics”, Oper. Theory Adv. Appl., 104, pp. 37-47, Birkhäuser, Basel, (1998)

[12] Biane, P.: On the free convolution with a semi-circular distribution. Indiana Univ. Math. J. **46**(3), 705–718 (1997)

[13] Biane, P.: Processes with free increments. Math. Z. **227**(1), 143–174 (1998)

[14] Bożejko, M., Bryc, W.: On a class of free Lévy laws related to a regression problem. J. Funct. Anal. **236**(1), 59–77 (2006)

[15] Bożejko, M., Leinert, M., Speicher, R.: Convolution and limit theorems for conditionally free random variables. Pacific J. Math. **175**(2), 357–388 (1996)

[16] Bun, J., Allez, R., Bouchaud, J.-P.: Potters M. Rotational Invariant Estimator for General Noisy Matrices,: IEEE Transactions on Information Theory **62**, 12 (2016)

[17] Chang, J.T., Pollard, D.: Conditioning as disintegration. Stat. Neerl. **51**, 287–317 (1997)

[18] Connes, A.: Classification of injective factors. Cases . Ann. of Math. (2) **104**(1), 73–115 (1976)

[19] Dellacherie, C., Meyer, P.-A.: Probabilities and potential, North-Holland (1978)

[20] Ji, H.C.: Regularity properties of free multiplicative convolution on the positive line. Int. Math. Res. Not. IMRN **6**, 4522–4563 (2021)

[21] Ledoit, O., Péché, S.: Eigenvectors of some large sample covariance matrix ensembles. Probab. Theory Related Fields **151**(1–2), 233–264 (2011)

[22] Lehner, F.: Szpojankowski, K. Free integral calculus I. arXiv:2311.04039

[23] Mingo, J.A., Speicher, R.: Free probability and random matrices, Springer Verlag (2017)

[24] Nica, A., Speicher, R.: Lectures on the combinatorics of free probability, Cambridge University Press (2006)

[25] Potters, M., Bouchaud, J.-P.: A first course in random matrix theory, Cambridge University Press (2021)

[26] Robbins, H.: An empirical Bayes approach to statistics. Proceedings of the Third Berkeley Symposium on Mathematical Statistics and Probability, 1954–1955, vol. I, pp. 157–163, University of California Press, (1956)

[27] Semerjian, G.: Matrix denoising: Bayes-optimal estimators via low-degree polynomials. arXiv:2402.16719

[28] Takesaki, M.: Theory of operator algebras. I, Springer Verlag (1979)

[29] Torchinsky, A.: Real-variable methods in harmonic analysis, Dover Publications (2004)

[30] Zhong, P.: On the free convolution with a free multiplicative analogue of the normal distribution. J. Theoret. Probab. **28**(4), 1354–1379 (2015)

